# Evaluation of epistasis detection methods for quantitative phenotypes

**DOI:** 10.1093/bioinformatics/btag344

**Published:** 2026-06-04

**Authors:** Stanislav Listopad, Gauri Renjith, Qian Peng

**Affiliations:** Department of Neuroscience, The Scripps Research Institute, La Jolla, CA 92037, United States; Department of Computer Science and Engineering, University of California, San Diego, CA 92093, United States; Department of Neuroscience, The Scripps Research Institute, La Jolla, CA 92037, United States

## Abstract

**Motivation:**

Epistasis, or genetic interaction, plays a crucial role in shaping complex traits and has been increasingly recognized for its widespread influence in genetic architectures. While epistasis detection has been extensively evaluated in case-control studies, its performance with quantitative phenotypes remains comparatively understudied.

**Results:**

We identified and evaluated six epistasis detection methods applicable to quantitative trait analysis: EpiSNP, Matrix Epistasis, MIDESP, PLINK Epistasis, QMDR, and REMMA. Using the EpiGEN simulator, we generated synthetic datasets modeling four classes of pairwise SNP interactions—dominant, multiplicative, recessive, and XOR. We also assessed BOOST and MDR algorithms using discretized (case-control) versions of the same datasets. Performance varied notably by interaction type: REMMA achieved the highest overall detection rate (55%), particularly excelling with dominant interactions (100%). MDR excelled with multiplicative (57%) and XOR (69%) interactions. Meanwhile, EpiSNP attained the best performance for recessive interactions (67%). All methods except BOOST produced *F1* scores below 0.05 for most interaction types. We further evaluated the methods using a real-world dataset. When applied to the Adolescent Brain Cognitive Development dataset to analyse the externalizing behavior phenotype, both PLINK Epistasis and PLINK BOOST identified SNPs within the *DRD2* and *DRD4* genes, consistent with previously reported genetic associations. Given the variability in tool performance across interaction types, no single method provides optimal detection across all scenarios. Leveraging multiple detection algorithms may therefore yield more comprehensive insights into epistatic effects in quantitative trait analyses.

**Availability and implementation:**

All relevant code and simulated datasets can be found at github.com/staslist/Epistasis_Review repository.

## 1 Introduction

Over a decade of genome-wide association studies (GWASs) have generated robust results for a broad range of complex traits and diseases with increasingly large sample sizes (Corvin *et al.* 2010, Uffelmann *et al.* 2021). While GWAS is a powerful tool for genetic mapping, most variants have shown small effects and captured a fraction of the disease heritability in general. The gap between the phenotypic variations that can be explained by genetic variants identified by GWAS and that were estimated by twin and family studies is referred to as “missing heritability” (Manolio *et al.* 2009, Singhal *et al.* 2023, Burch *et al.* 2024). A number of factors have been hypothesized to account for missing heritability. Genetic interaction is one such potentially critical culprit (Eichler *et al.* 2010). The present association studies model genetic effects of multiple variants in a linear combination (assuming additivity), while combinatorial effects beyond the linear effects are not considered. When phenotypic effects from two genetic variants deviate from the expected additive value of the individual mutations, the two variants are said to exhibit an epistatic interaction (Fisher 1919). Once treated as an exception, epistasis has been increasingly recognized as ubiquitous (Kauffman 1993, Moore 2003). It has been shown that disease genes have a high propensity to interact with each other (Barabási *et al.* 2011). Additionally, the majority of genetic variants associated with complex diseases are located in non-coding regions, suggesting that they may influence traits through regulatory interactions (Pickrell 2014).

Although epistasis has not been systematically considered in most genetic studies, evidence suggests that it contributes to susceptibility to common human diseases, such as Alzheimer’s (Moore 2003, Ebbert *et al.* 2015, Abd El Hamid *et al.* 2023, Zhang *et al.* 2024). The term epistasis may refer to biological or statistical epistasis (Cordell 2002, Niel *et al.* 2015). Biological epistasis involves physical interaction between two or more biological components. For instance, hair color in humans is influenced by such epistasis: the interaction between *MC1R* and *ASIP* genes is partially responsible for red hair color (Morgan *et al.* 2018). Meanwhile, statistical epistasis refers to the departure from additive effects of genetic variants at different loci with regard to their global contribution to the phenotype. Biological epistasis can be modeled by statistical epistasis, although statistical interaction does not always imply biological interaction. Elucidating biological interaction from statistical one is a challenging task. Improving the quality of statistical epistasis detection is nonetheless a crucial step to enable the discovery of more biological interactions. These biological interactions, may then in turn, improve our understanding of heritable traits. Given the availability of large GWAS datasets and a number of epistasis detection methods that have been developed over the years, statistical epistasis analyses may be potentially carried out at large scales. Several publications have compared the performance of epistasis detection methods primarily suitable for case-control data (Wang *et al.* 2011, Chatelain *et al.* 2018, Ponte-Fernández *et al.* 2022, Russ *et al.* 2022, Ren and Liang 2024). However, less is known about the performance of epistasis detection tools suitable for quantitative phenotypes. Quantitative traits are continuous measurable phenotypes that are influenced by a combination of genes and environment. Quantitative traits are extremely common, with some examples being: height, weight, blood pressure, Bilirubin (on metabolic panel), etc. It is impractical to study living organisms without interacting with quantitative phenotypes. To this end, we conducted a survey of the commonly used epistasis detection tools, evaluating their performance on a set of simulated data, followed by testing on a real-world dataset.

For this study, we used EpiGEN to generate simulated epistasis datasets with quantitative phenotypes (Blumenthal *et al.* 2020). Given the combinatorial nature of genetic interactions, many types of epistasis theoretically exist. We modeled four major types of interactions that are most plausible: dominant, multiplicative, recessive, and exclusive-or (XOR). In the dominant model, as defined by EpiGEN, an interaction occurs if both single nucleotide polymorphisms (SNPs) each have at least one minor allele. In the multiplicative model, an interaction occurs if either SNP carries at least one minor allele, with the strength of the interaction increasing proportionally to the number of minor alleles present in the interacting SNPs. In the recessive model, an interaction is observed only if both SNPs have two minor alleles. In the XOR model, an interaction occurs only if one of the SNPs, has at least one minor allele, but not both. Note that other definitions of XOR epistasis exist, such as the one defined by Sha *et al.* (2024). While the dominant, recessive, and XOR interactions are considered biologically plausible (Batista *et al.* 2023, Sha *et al.* 2024). There is no clear evidence establishing the existence of multiplicative interactions, to the best of our knowledge. However, the multiplicative interaction is often used as an approximation for various types of interactions, especially when the exact nature of the interactions is uncertain. Throughout this publication, we use the EpiGEN-specific definitions of dominant, multiplicative, recessive, and XOR epistasis, as these terms may have alternative meanings in broader genetic contexts.

After generating the simulated data, we identified several publicly available epistasis detection tools suitable for analysing quantitative phenotype data, including EpiSNP, Matrix Epistasis, MIDESP, PLINK Epistasis, QMDR, and REMMA (Purcell *et al.* 2007, Ma *et al.* 2008, De *et al.* 2015, Zhu and Fang 2018, Wang *et al.* 2020, Heinrich *et al.* 2021). Our primary goal was to examine tools that could directly analyse quantitative phenotype data, which, when available, usually offers increased statistical power for detection. However, we also recognize that in certain settings, discretizing the phenotype into a binary (case-control) outcome may be viable, thus enabling the use of a broader set of analysis methods. Accordingly, we evaluated the Boolean operation-based screening and testing (BOOST) algorithm implemented in PLINK and the multifactor dimensionality reduction (MDR) algorithm implemented in QMDR to test whether phenotype discretization can serve as a viable strategy (Ritchie *et al.* 2001, Wan *et al.* 2010). A complete list of tools we attempted to evaluate is provided in Table 1. While our coverage of tools is not exhaustive, we aimed to include tools that employ some of the most used epistasis detection models, such as linear regression and linear mixed models (LMMs), among others. We then assessed the performance of each tool in detecting dominant, recessive, multiplicative, and XOR interactions.

**Table 1:** List of tools evaluated, their underlying epistasis models, and their evaluation statuses.^a^

Name	Underlying model	Simulated data	ABCD data
EpiSNP (Ma *et al.* 2008)	General linear model	✓	Errors during execution
FastANOVA (Zhang *et al.* 2008)	ANOVA	Failed installation	Not applicable
FRGEpistasis (Zhang *et al.* 2014)	Functional regression model	Can only perform gene x gene interaction testing, not SNP × SNP	Not applicable
Matrix Epistasis (Zhu and Fang 2018)	Linear regression	✓	✓
MIDESP (Heinrich *et al.* 2021)	Mutual information	✓	✓
PLINK (BOOST and Epistasis) (Purcell *et al.* 2007, Wan *et al.* 2010)	BOOST, linear regression	✓	✓
QMDR (Ritchie *et al.* 2001, De *et al.* 2015)	MDR	✓	✓
REMMA (Wang *et al.* 2020)	Linear mixed model	✓	✓
SNPassoc (González *et al.* 2007)	Log likelihood ratio test	Successfully used with <200 SNPs, but could not use with 1000 SNPs	Not applicable
WISH-R (Carmelo *et al.* 2018)	Linear regression	Failed installation	Not applicable

a Underlined tools were further evaluated in the study. Checkmarks (✓) indicate successful installation and usage.

Epistasis can involve interactions between two or more SNPs, but this review focuses exclusively on pairwise interactions. Interaction between two SNPs is referred to as second-order epistasis, while interaction between more than two SNPs is referred to as higher-order epistasis. There are two categories of epistasis detection methods: exhaustive and non-exhaustive. Exhaustive algorithms test every possible combination of SNPs, which can quickly become unsustainable for higher-order epistasis as the number of comparisons grows exponentially with the order of epistasis. The huge number of comparisons then results in lengthy runtime and low statistical power. Non-exhaustive epistasis detection methods mitigate these challenges by evaluating only a subset of all possible comparisons. However, there is evidence suggesting that they may perform worse than exhaustive methods (Ponte-Fernández *et al.* 2022). For these reasons, this study focuses on second order epistasis detection methods.

In addition to using simulated data, we evaluated the tools in this study using the Adolescent Brain Cognitive Development (ABCD) dataset. Unlike simulated data, ABCD dataset includes population structure, individual relatedness, and multiple covariates, which add complexity to the analysis. It also contains a larger number of samples and SNPs compared to our simulated datasets. Therefore, the ABCD dataset provided a valuable opportunity to compare the performance of epistasis detection tools in a real-world context. For this study, we focused on the externalizing behavior phenotype, which is both heritable and common (Maes *et al.* 2023).

## 2 Materials and methods

### 2.1 Data generation

#### 2.1.1 Quantitative phenotype datasets

A total of 56 datasets were generated using EpiGEN (Blumenthal *et al.* 2020), each containing a single type of epistatic interaction: dominant, recessive, multiplicative, or XOR. Within each dataset, all interactions had the same interaction alpha (Blumenthal *et al.* 2020). Interaction alpha is a positive number that quantifies interaction strength. Definitions of dominant, recessive, and multiplicative interactions in terms of interaction alpha can be found in the EpiGEN publication. The XOR was defined as follows: if one SNP genotype is in {1, 2} (at least one minor allele) and the other SNP genotype is in {0} (no minor alleles) then there is an interaction. There are 20 multiplicative, eight recessive, 24 dominant, and four XOR datasets. All interactions occurred between pairs of disease SNPs. Forty datasets contained 1000 SNPs and 1000 individuals, while 16 larger datasets contained up to 5000 SNPs and 5000 individuals, with the data generated using chromosome one of the CEU HapMap3 cohort (Gibbs *et al.* 2003). While these datasets are much smaller than those used in a typical GWAS, they possess some (but not all) of the same key properties as real SNP data such as linkage disequilibrium (LD).

Out of the 20 multiplicative datasets, 16 contained five disease SNPs and four contained 20 disease SNPs. The datasets with five disease SNPs were created by permuting the following variables: interaction alpha {1.25, 1.5, 2, 3}, number of interacting SNP pairs {1, 2}, and purity status {pure, impure}, resulting in 16 datasets. Lower interaction alphas such as 1.25 and 1.5 produce phenotype values for individuals with interacting disease SNPs that are just a few times larger than those without interacting disease SNPs, making detection challenging. Larger interaction alphas such as two and three lead to phenotype values for individuals with interacting disease SNPs to be tens of times larger than phenotype values of individuals without interacting disease SNPs, making these interactions easier to detect. Pure datasets contained no individual main effects, while impure datasets included some individual main effects (Russ *et al.* 2022). The individual main effects in impure datasets were implemented using recessive, additive, and dominant marginal models (File S1, available as supplementary data at *Bioinformatics* online). The four datasets with 20 disease SNPs were created by permuting interaction alphas {1.25, 1.5} and purity status {pure, impure}, with each dataset containing eight interacting disease SNP pairs. In total, the 20 multiplicative datasets contained 56 interacting SNP pairs (8 * 1 + 8 * 2 + 4 * 8 = 56).

The 24 dominant datasets were divided into two categories: eight regular dominant datasets, consisting of 1000 SNPs and 1000 individuals, and 16 large dominant datasets consisting of up to 5000 SNPs and 5000 individuals. The large dominant datasets were designed to evaluate the impact of SNP count, sample size, and disease-associated SNP minor allele frequency (MAF) on epistasis detection. The eight regular dominant and eight recessive datasets each contained five disease-associated SNPs. These datasets were created by permuting interaction alpha values {8, 16}, the number of interacting SNP pairs {1, 2}, and purity status {pure, impure}. The eight dominant and eight recessive datasets contained a total of 24 interacting SNP pairs (4 * 1 + 4 * 2 + 4 * 1 + 4 * 2 = 24). All 16 large dominant datasets contained five disease SNPs, two interacting SNP pairs, and were impure. Half of the large dominant datasets had interaction alpha value of eight and half had interaction alpha value of 16. The large dominant datasets contained a total of 32 interacting SNP pairs. The four XOR datasets each contained 20 disease SNPs with eight interacting SNP pairs, resulting in a total of 32 interacting SNP pairs across the XOR datasets. These datasets were created by permuting interaction alphas {1.5, 2} and purity status {pure, impure}. Interaction alphas of eight and 16 were chosen to ensure that the phenotypes of individuals with interacting disease SNPs were distinct from those without interacting disease SNPs. Full in-depth specifications used to generate each dataset can be found in File S1, available as supplementary data at *Bioinformatics* online.

#### 2.1.2 Case-control datasets

All of the above 56 datasets were also discretized to create two additional sets of 56 case-control datasets each (a total of 112 case-control datasets). The discretization thresholds were selected individually for each dataset to ensure a reasonable balance of cases and controls, typically resulting in at least one % of samples being cases. The first set of binarization thresholds was conservative, such that only phenotypes with strong disease SNP interactions would be labeled as cases. The second set of binarization thresholds was uniform for all datasets, such that any phenotype 50% larger than the baseline would be labeled as case. This resulted in much larger number of cases (Table 2). While discretizing the phenotype results in a loss of information, it enables the use of a much larger number of tools that are suitable for case-control epistasis analysis, compared to tools designed for quantitative epistasis analysis. Further details on the discretization process can be found in File S1, available as supplementary data at *Bioinformatics* online.

**Table 2:** Number of case-controls for each dataset type.^a^

Dataset type	# of individuals with detectable interacting SNP pair(s)	Threshold #1: number of cases/controls	Threshold #2: number of cases/controls
Recessive	2.6	6.4/993.6	178.5/821.5
Regular dominant	17.7	17.9/982.1	80.2/919.8
Large dominant	230.1	227.1/3022.9	633.5/2616.5
Multiplicative	433.2	92.9/907.1	323.7/676.3
XOR	863.25	481.7/518.3	752.0/248

a The number of individuals with detectable interacting SNP pair(s) is provided as a reference for number of cases. The binarization thresholds #2 are strictly smaller than binarization thresholds #1 and result in strictly larger number of cases.

### 2.2 ABCD dataset

The ABCD study is an ongoing project aimed at studying brain development and child health (Karcher and Barch 2021). One of the more highly heritable quantitative phenotypes in the study is externalizing behavior (Maes *et al.* 2023), which comprises of a wide range of antisocial behaviors such as aggression, bullying, conduct problems, rule breaking, and substance use (Kauten and Barry 2020, Vergunst *et al.* 2023, Petersen 2024). Due to its common occurrence among children, externalizing behavior is an ideal phenotype for study. Additionally, some genetic interactions associated with externalizing behavior, such as *DRD4*–*DRD2* and *DRD4*–*SLC6A4* interactions, have already been identified (Beaver *et al.* 2007, Hohmann *et al.* 2009, Koyama *et al.* 2024). *DRD4* and *DRD2* are dopamine receptor genes, while *SLC6A4* is a serotonin transporter gene. The ABCD study contains various externalizing scores. For this analysis, we used the child behavioral check list (CBCL) externalizing *t*-score, with *t*-score >64 indicating clinical symptoms (Guerrera *et al.* 2019).

The cohort includes 11 666 children, of which 417 had externalizing *t*-score >64, after excluding 202 individuals due to missing principal components of kinship matrix. The participants come from different ancestry backgrounds and exhibit some degree of relatedness. The externalizing *t*-scores were normalized for age and sex, then adjusted for population stratification (using the top 10 principal components of the kinship matrix) and batch site covariates (Balvert *et al.* 2024); the resulting residuals were used in subsequent analyses. For REMMA, which accounts for population stratification and relatedness using LMM, only batch site covariate was used to obtain the residuals. While some of the tools in the study, such as Matrix Epistasis, can directly account for covariates, we opted to use residuals to ensure uniformity across all tools. The discretized phenotypes were obtained by converting the residuals into case-controls with a threshold of 20.4, chosen to match the ratio of cases to controls when using the original *t*-score threshold of 64. Genetic data were obtained using hg19 smokescreen array as documented in the ABCD study (Baurley *et al.* 2016), with 515 279 SNPs in the dataset. Initially, we intended to perform epistasis analysis on the entire dataset, but only PLINK was able to complete the analysis within 24 h. Therefore, we confined the analysis to SNPs on chromosome 11, which includes 24 699 SNPs. Chromosome 11 contains the *DRD4* and *DRD2* genes, as well as a few other genes linked to externalizing behavior, such as *NCAM1*, *BDNF*, and *TPH1* (Arcos-Burgos *et al.* 2012, Cecil *et al.* 2018, Xiang *et al.* 2019). Limiting the analysis to a smaller number of SNPs allowed us to include all tools except EpiSNP in the comparative analysis (Table 1). EpiSNP encountered execution errors, likely due to the large dataset size. The data contained 446 SNPs with missing values. PLINK (Epistasis and BOOST), MIDESP, and REMMA were used with missing data as is. For other tools, missing SNPs were imputed with the most common non-missing values. Detailed information regarding the genetic and externalizing phenotype data can be found in the ABCD study release notes website.

A Bonferroni correction was applied to account for multiple testing, setting the *P-*value significance threshold at 1.64e−10 for the analysis of 24 699 SNPs. The Bonferroni correction tends to be overly conservative since the SNP-SNP interactions are correlated. Hence, a suggestive significance threshold of 1.64e−9 was also used. For tools that did not provide *P-*value, such as MIDESP, MDR, and QMDR, we retained the top 0.001% of reported results for MIDESP or the top 500 results for MDR and QMDR, based on their respective evaluation metrics (mutual information [MI], *t*-statistic, and balanced accuracy). While in practice additional steps could be taken to establish the significance of the results reported by these tools, the goal of this study was to evaluate the tools based on their direct output. The interacting SNPs were then mapped onto genes using the UCSC hg19 gene annotation.

### 2.3 Epistasis tool selection

We began by conducting an extensive search for epistasis detection tools that could analyse quantitative phenotype data. We focused on publications offering ready-to-use software packages with available documentation. We identified 10 tools that were accessible online (Table 1). Since linear regression model is widely used, we evaluated multiple tools based on this model. For other epistasis detection models, we chose the most representative tool for evaluation. After several attempts to install the tools on a personal computer and a high-performance computing (HPC) cluster, we decided to evaluate EpiSNP, Matrix Epistasis, MIDESP, PLINK Epistasis, QMDR, and REMMA. These tools were well documented, simple to install and use, and represented a diverse set of epistasis detection models. PLINK supports multiple epistasis models, and we used its linear regression model for quantitative datasets and its BOOST implementation for case-control datasets. Similarly, we applied QMDR to both quantitative and case-control datasets. We selected BOOST and QMDR for analysis of discretized data, based on previous reviews of epistasis detection algorithms (Russ *et al.* 2022). For simplicity, we refer to QMDR as MDR when applied to binary phenotype data. All selected tools were well optimized and capable of analysing >1000 SNPs at a time.

### 2.4 Epistasis detection models

Linear regression is a linear model used to predict the value of a scalar independent variable based on dependent variable(s). It is used as the underlying model in many epistasis detection tools due to its ability to approximate various types of interactions while being easy to implement and use. The model is similar to the linear regression used in GWAS, except that it includes a term representing SNP–SNP interaction. This allows for a direct interpretation of the results (Cordell 2002). Both PLINK Epistasis and Matrix Epistasis use this model to estimate interaction coefficients for each SNP pair. The key advantages of this model are its simplicity, interpretability, and fast execution. However, it has limitations, including the assumption of linear relationship between variables and its susceptibility to outliers.

LMM is an extension of linear regression that allows for both fixed and random effects. LMMs offer significant advantages over traditional linear regression, particularly in genetic studies, as they can account for population stratification and individual relatedness (Wang *et al.* 2020). The increased complexity of LMM, however, results in longer execution times compared to simpler models like linear regression. REMMA employs the linear mixed model in epistasis detection.

General linear model (GLM) is a broad class of models that includes linear regression, analysis of variance (ANOVA), and analysis of covariance (ANCOVA) (Nelder and Wedderburn 1972). Linear regression is a specific case of GLMs. EpiSNP utilizes GLMs to approximate epistasis, which is similar to, but not identical to, the approach used by PLINK and Matrix Epistasis. The advantages and disadvantages of GLMs are largely similar to those for linear regression.

MI model is based on information theory. It quantifies the amount of information obtained about one random variable by observing another. MI has a wide variety of applications (Heinrich *et al.* 2021). MIDESP computes the amount of MI shared between a subset of most promising SNP pairs and the phenotype. As a feature selection method, MI does not require any prior assumptions and can be used for both categorical and continuous data. However, its main drawback is that it can be computationally expensive to compute.

MDR model is a dimensionality reduction approach for detecting interactions between independent variables influencing a dependent variable (Ritchie *et al.* 2001). For a pair of biallelic SNPs, MDR considers nine possible genotype combinations: 0–0, 0–1, 0–2, 1–0, 1–1, 1–2, 2–0, 2–1, and 2–2 (numbers represent the number of minor alleles). For each of these combinations, a ratio of cases to controls is calculated. Genotypes where cases outnumber controls are labeled as high risk, while the opposite are labeled as low risk. Thus, a pair of SNPs, is represented as a feature with two values: low risk and high risk. A machine learning (ML) technique is then applied to identify how useful each feature is for distinguishing between cases and controls. QMDR extends this algorithm to quantitative phenotypes, by comparing mean phenotype values instead of case-control ratios. MDR transforms the data and combines it with a ML classifier to detect epistasis. The strength and weakness of this approach depend on the choice of ML classifier used.

### 2.5 Data wrangling

The JSON files generated by EpiGEN had to be transformed into appropriate format for each of the selected epistasis detection tools. Several tools (MIDESP, Plink, and REMMA) use standard PLINK input files, while the rest require custom formatting.

### 2.6 Performance evaluation

All of the selected epistasis detection tools returned lists of interacting SNP pairs. EpiSNP, Matrix Epistasis, PLINK (Epistasis and BOOST), and REMMA provided *P-*value for each SNP pair, while MIDESP provided MI values. QMDR provided balanced accuracy values for binary phenotype data, and *t*-statistic for quantitative phenotype data. We used these values to sort the SNP pairs from most to least significant. *P-*value were sorted in ascending order, while the other metrics were sorted in descending order. Each tool had a mechanism for limiting the number of output SNP pairs. For tools that used *P-*value as the sorting metric, a reporting threshold was computed using Bonferroni correction. As an example, for a 1000 SNP dataset the threshold would be 0.05 * 2/1000^2^ = 1e−7. MIDESP reported the top 1% of hits by default, and MDR and QMDR reported up to top the 500 hits. Full details regarding the runtime configuration of each tool can be found in File S2, available as supplementary data at *Bioinformatics* online.

The sorted lists of hits were then assessed based on three criteria: true positives, the average ranking of the true positives, and the *F*1 score. The average ranking of the true positives was computed using two approaches: penalized and unpenalized. In the penalized approach, for datasets with more than one interacting SNP pair, any undetected pairs were considered to be at the end of the sorted hit list. In unpenalized approach, undetected pairs were ignored. In datasets with zero true positive, the average true positive ranking was considered not applicable.

## 3 Results

### 3.1 True positives detected

**Figure 1:**
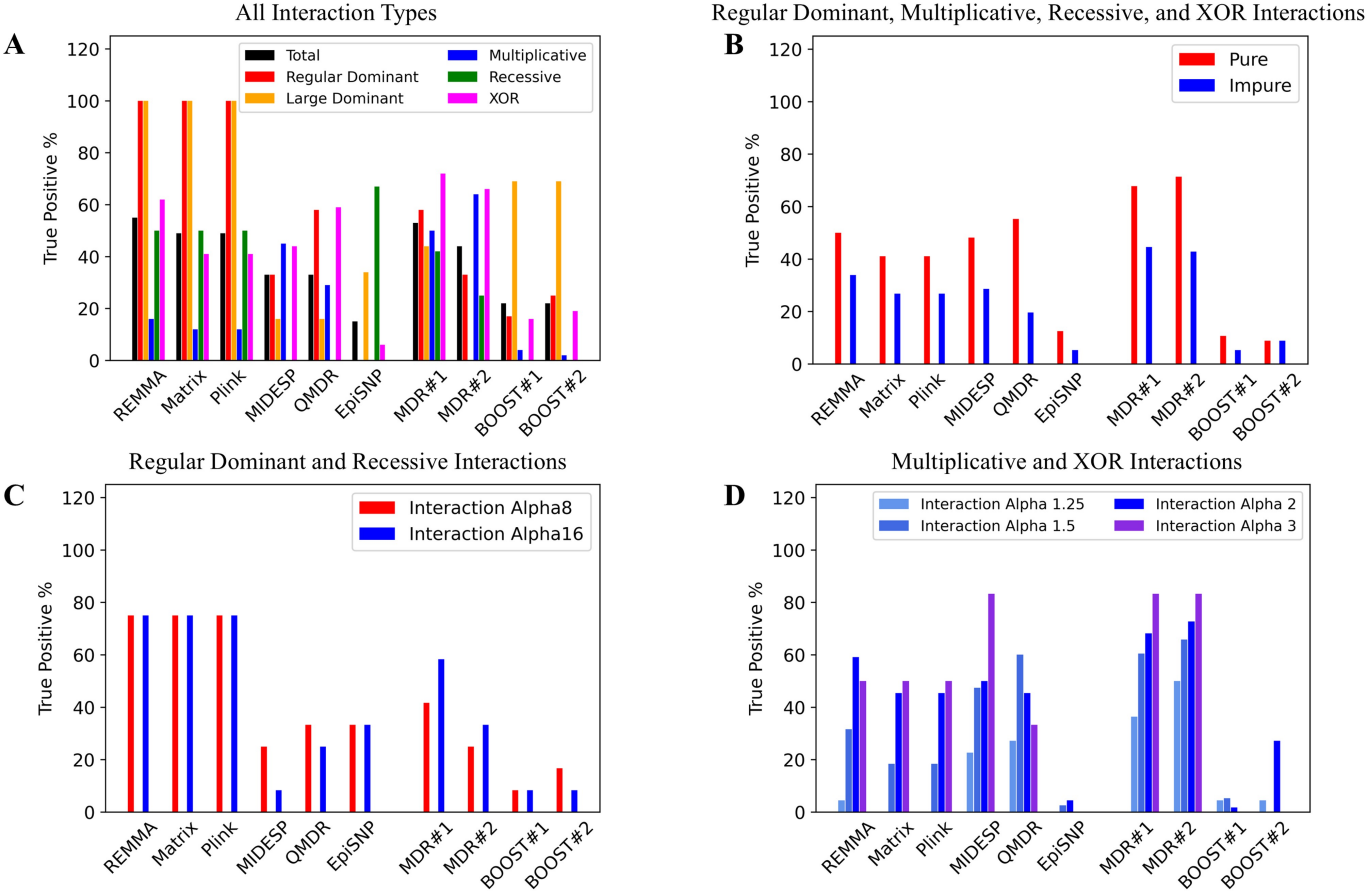
The number of successfully detected interacting SNP pairs (true positive %) by tools and epistasis interaction types. (A) Overall performance of each tool for each interaction type. (B) Pure versus impure datasets. (C) Regular dominant and recessive datasets sub-grouped by interaction alpha values: 8 and 16. (D) Multiplicative and XOR datasets sub-grouped by interaction alpha values: 1.25, 1.5, 2, and 3. Note that large dominant datasets are excluded from subplots B, C, and D.

We examined the number of true positives detected by each tool based on the type of interaction in the EpiGEN simulated data (Table 3, Fig. 1). The true positive percentage (%), also known as sensitivity, refers to the number of successfully detected interacting SNP pairs divided by the total number of truly interacting SNP pairs present in the data. Matrix Epistasis, PLINK Epistasis, and REMMA exhibited nearly identical results, as expected, due to their similar underlying models. These tools excelled at detecting dominant epistasis interactions, identifying 100% of the true positives. REMMA exhibited the highest overall sensitivity of 55%. MIDESP demonstrated a drastically different performance profile compared to Matrix Epistasis, PLINK Epistasis, and REMMA. It was unable to detect recessive interactions and only identified 20% of the dominant interactions. However, MIDESP performed better at detecting multiplicative and XOR interactions, with detection rates of 45% and 44%, respectively. QMDR demonstrated the same overall sensitivity of 33% as MIDESP with a similar performance profile across various interaction types. EpiSNP performed best for recessive interactions, with a 67% detection rate, but performed poorly with other interaction types.

**Table 3:** True positive rate (sensitivity) for each tool overall and grouped by interaction type.^a^

Tool		Sensitivity %					
	Total	Reg. Dom.	Large Dom.	(Reg. & Large) Dom.	Mul.	Rec.	XOR
MIDESP	33	33	16	20	45	0	44
EpiSNP	15	0	34	25	0	67	6
Matrix	49	100	100	100	12	50	41
PLINK	49	100	100	100	12	50	41
REMMA	55	100	100	100	16	50	62
QMDR	33	58	16	27	29	0	59
BOOST#1	22	17	69	54	4	0	16
BOOST#2	22	25	69	57	2	0	19
MDR#1	53	58	44	48	50	42	72
MDR#2	44	33	0	9	64	25	66

a The interaction types are: regular dominant (Reg. Dom.), large dominant (Large Dom.), multiplicative (Mul.), recessive (Rec.), and XOR. The dominant datasets are subdivided into regular dominant datasets and large dominant datasets. The true positive rate for all dominant datasets combined is listed in the (regular & large) dominant datasets column.

BOOST and MDR cannot analyse quantitative phenotype data directly. Instead, they were ran with the two sets of case-control datasets that were generated by discretizing the quantitative datasets using two different thresholds (Table 2). MDR’s performance varied greatly depending on the binarization threshold used. Specifically, MDR tended to perform better when the number of cases more closely matched the number of samples with detectable interaction (Tables 2 and 3). This makes sense intuitively as an interacting SNP pair is a good classification feature only if cases are chiefly defined by presence of this SNP-SNP interaction. MDR’s overall performance with the first set of case-control datasets (53%) was nearly as good as that of REMMA. However, its overall performance degraded with the second set of case-control datasets (44%), wherein many of the cases contained marginal, but no interaction effects. MDR performed especially well with multiplicative (50% with threshold set #1; 64% with threshold set #2; 57% average) and XOR (72% with threshold set #1; 66% with threshold set #2; 69% average) datasets. BOOST, on the other hand, exhibited similar performance with both sets of case-control datasets. BOOST exhibited low overall sensitivity of 22% (with both sets of case-control datasets), being the second to last tool when ranked by overall sensitivity.

### 3.2 Average true positive ranking

**Figure 2:**
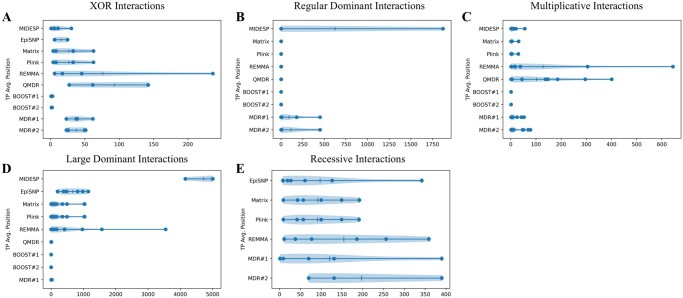
The distribution of unpenalized true positive rankings is shown as violin charts for each epistasis detection tool, categorized by interaction type. (A) Average true positive ranking for XOR datasets. (B) Average true positive ranking for regular dominant datasets. (C) Average true positive ranking for multiplicative datasets. (D) Average true positive ranking for large dominant datasets. (E) Average true positive ranking for recessive datasets. Tools omitted in each subplot failed to make any detections.

While the number of successful true positive detections is important, such detections are more valuable if they are ranked highly on the sorted list of pairwise SNPs (i.e. the most significant). Otherwise, distinguishing true positives from false positives becomes challenging. Therefore, for each tool, for each dataset, we calculated the unpenalized average true positive ranking (the average ranking across all interacting pairs within a single dataset) based on the interaction type (Fig. 2). Datasets where no true positives were detected were excluded from this analysis. For all interaction types, except recessive, most true positives were ranked toward the top of the respective sorted lists. The mean and median unpenalized average true positive rankings for each tool and interaction type are summarized in Table 4.

**Table 4:** Summary of the mean and median unpenalized average true positive (TP) ranking for each tool and interaction type.^a^

Tool	Mean; median TP position				
	Reg. Dom.	Large Dom.	Mul.	Rec.	XOR
MIDESP	624; 1	4719; 4996	**7; 3**	NA; NA	12; **8**
EpiSNP	NA; NA	671; 652	NA; NA	97; 44	15; 15
Matrix	**1; 1**	204; 31	**6; 1**	92; 78	27; 20
PLINK	**1; 1**	204; 31	**6; 1**	91; 78	27; 20
REMMA	**2; 1**	429; **8**	128; 16	154; 131	76; 31
QMDR	**1; 1**	**3; 1**	102; 25	NA; NA	93; 102
BOOST#1	**1; 1**	**2; 1**	**1; 1**	NA; NA	**2; 2**
BOOST#2	**1; 1**	**2; 1**	**1; 1**	NA; NA	**2; 2**
MDR#1	92; **1**	**7; 5**	12; **3**	120; 70	40; 38
MDR#2	115; **4**	NA; NA	16; **4**	197; 131	37; 38

a The means and medians are listed in the following order: regular dominant (Reg. Dom.), large dominant (Large Dom.), multiplicative (Mul.), recessive (Rec.), and XOR. If no true positives were identified for a given interaction type, the mean/median are marked as not applicable. Values below 10 are bolded.

For multiplicative interactions MIDESP shows high detection rates and high median average true positive rankings. For dominant interactions, Matrix Epistasis, PLINK Epistasis, and REMMA stand out as the top performers, exhibiting perfect detection rates and average true positive placements. For recessive interactions, EpiSNP is arguably the best tool with the highest detection rate and the best median average true positive placement. For XOR interactions, MIDESP stands out as having the highest mean and median average true positive rankings.

Regarding case-control methods, BOOST stands out as it tended to report all correctly identified interactions as the most significant interactions detected. This was the case for both sets of case-control datasets. MDR performed relatively well with multiplicative and XOR datasets, but was otherwise unremarkable.

Figure 2 and Table 4 can also be used to examine the impact of dataset size on average true positive placement in dominant datasets. As expected, larger datasets have more false positives, which leads to larger average true positive placements. However, even in larger datasets a substantial number of true positives are still detected within the top 10 (Table 5).

**Table 5:** Comparison of unpenalized average true positive (TP) placements between regular dominant and large dominant datasets for each tool.^a^

Tool	True positive placements	
	Regular dominant	Large dominant
MIDESP	**1, 1,** 1871.5	4154.5, 4996.5, 5007
EpiSNP	NA	199.5, 391.5, 482, 821.5, 983.5, 1151.5
Matrix	**1, 1, 1, 1, 1.5, 1.5, 1.5, 1.5**	**1.5, 1.5, 1.5, 2, 2, 4.5, 10,** 31, 32, 50, 76, 146.5, 351, 492.5, 1027.5, 1038.5
PLINK	**1, 1, 1, 1, 1.5, 1.5, 1.5, 1.5**	**1.5, 1.5, 1.5, 2, 2, 4.5, 10,** 31, 32, 50, 76, 146.5, 351, 492.5, 1026, 1038.5
REMMA	**1, 1, 1, 1, 2, 2, 2, 3**	**1.5, 1.5, 2, 2, 2, 2, 2.5, 8.5, 8.5,** 77, 90.5, 174, 416, 969, 1570, 3544.5
QMDR	**1, 1, 1, 1, 1.5, 2**	**1, 1, 2, 10**
BOOST#1	**1, 1**	**1.5, 1.5, 1.5, 1.5, 1.5, 1.5, 1.5, 1.5, 2, 2, 2**
BOOST#2	**1, 1, 1**	**1, 1, 1.5, 1.5, 1.5, 1.5, 1.5, 1.5, 1.5, 2, 2.5, 3.5**
MDR#1	**1, 1, 1, 1, 7,** 181, 451	**3, 3, 3, 3.5, 4, 5, 5, 5, 5,** 12, 13, 26
MDR#2	**1, 1, 7**, 451	NA

a If no true positives were identified for a given interaction type, true positive placements are listed as not applicable. Values below 10 are bolded.

### 3.3 *F*1 score

To further examine the ratio of true positives, false positives, and false negatives, we computed the *F*1 scores for each dataset. The mean *F*1 scores, averaged by interaction type for each tool, are shown in Table 6. The means were computed using only datasets where at least one true positive was detected. As indicated by the low *F*1 values, all tools except for BOOST generated many false positives (number of false negatives was negligible and can be disregarded). That said the number of false positives can be greatly reduced by using stricter significance cutoffs and/or retaining smaller percentage of top results (depending on method used).

**Table 6:** Summary of mean *F*1 scores for each tool, for each interaction type.^a^

Tool	Mean *F1* score				
	Reg. Dom.	Large Dom.	Mul.	Rec.	XOR
MIDESP	0.00136	0.00008	0.03286	NA	0.04491
EpiSNP	NA	0.00052	NA	0.00110	0.02653
Matrix	0.01017	0.00417	0.01715	0.00148	0.14969
PLINK	0.00995	0.00410	0.01423	0.00145	0.14140
REMMA	0.00501	0.00165	0.00241	0.00103	0.12694
QMDR	0.00349	0.00125	0.00317	NA	0.01870
BOOST#1	0.16667	0.25298	0.10000	NA	0.20940
BOOST#2	0.29167	0.43201	0.05000	NA	0.25385
MDR#1	0.00349	0.00349	0.00555	0.00249	0.02264
MDR#2	0.00199	NA	0.00713	0.00150	0.02067

a Only datasets wherein at least one true positive was detected were included in the computation of the mean. Interactions for which no true positives were detected are listed as not applicable.

### 3.4 Impact of purity status and interaction alpha

For most tools and interaction types, the detection rate was higher in pure datasets without individual main effects. The impact of interaction alpha on detection rate varied across tools. The number of interactions detected by each tool, categorized by dataset purity status and interaction alpha value, are shown in Fig. 1B. A detailed breakdown of each tool’s performance by interaction alpha and dataset purity status can be found in File S3, available as supplementary data at *Bioinformatics* online.

### 3.5 Impact of SNP count, sample size and disease-associated SNP MAF

**Figure 3:**
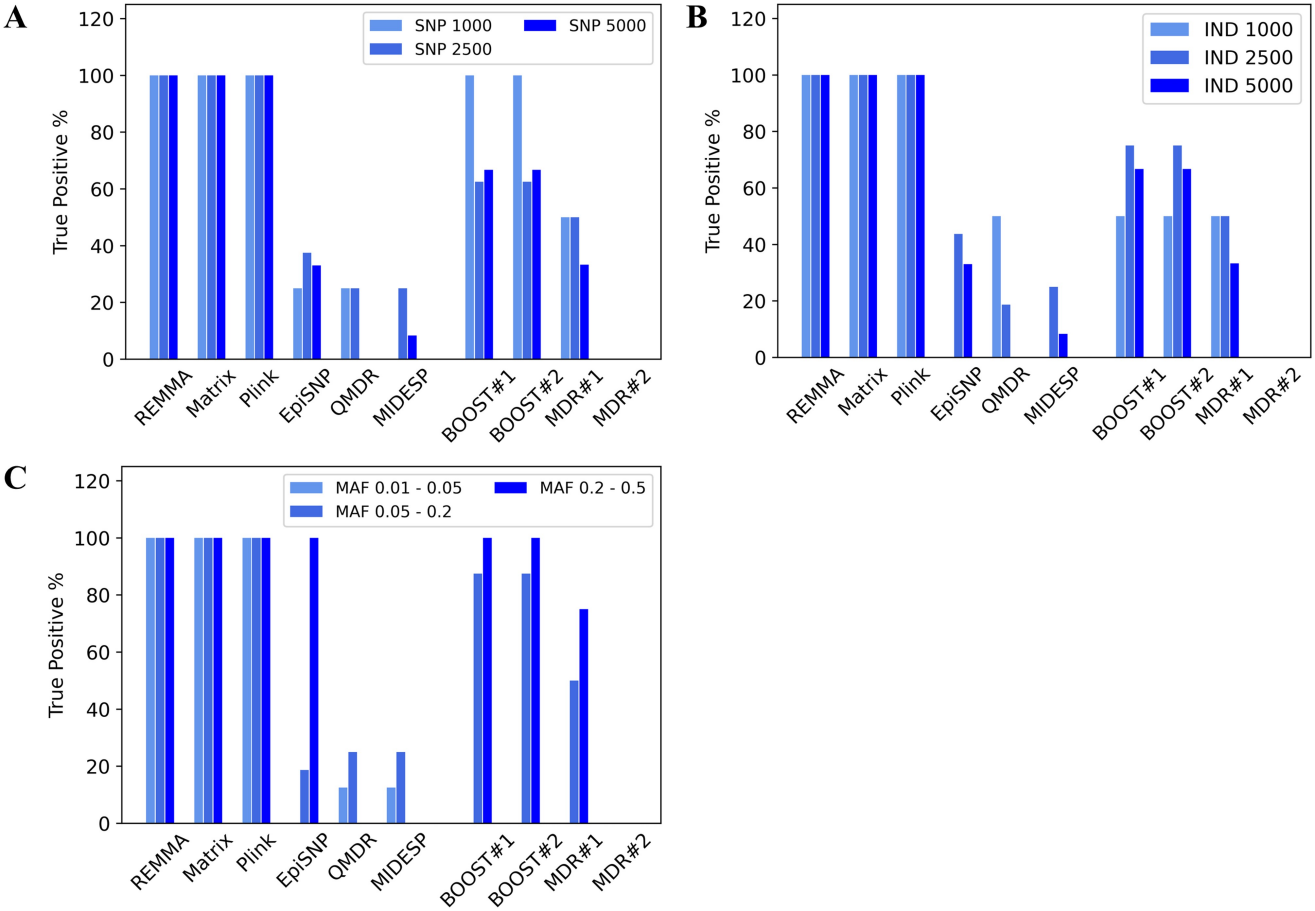
Impact of SNP count, sample size, and disease-associated SNP MAF on sensitivity in large dominant datasets. (A) True positive rate for each tool is grouped by the SNP count of the dataset. (B) True positive rate for each tool is grouped by the sample size of the dataset. (C) True positive rate for each tool is grouped by the minor allele frequency range of the disease-associated SNPs within each dataset.

The three tools that exhibited 100% sensitivity in regular dominant datasets showed identical sensitivity in the large dominant datasets. The remaining tools had lower true positive rates, and their performance varied depending on dataset size and minor allele frequency of the disease-associated SNPs (Fig. 3). Most tools performed slightly worse in datasets with larger SNP counts. In contrast, sample size did not show a consistent positive or negative impact on performance. An increase in the MAF of disease-associated SNPs was associated with higher true positive detection rate. This may be because datasets with larger MAF have a larger case-control ratio (Table 7).

**Table 7:** Case-control ratios in the large dominant datasets stratified by the MAF range of the disease-associated SNP.

	MAF: 0.01–0.05	MAF: 0.05–0.2	MAF: 0.2–0.5
# of cases (threshold #1)	5	101	701
# of controls (threshold #1)	3745	2649	3049
# of cases (threshold #2)	89	392	1662
# of controls (threshold #2)	3661	2358	2088

### 3.6 Runtime

We briefly evaluated the runtimes of PLINK Epistasis across a range of SNP and sample sizes. PLINK Epistasis was chosen for this evaluation because it is one of the most highly optimized tools included in the study. All analyses were executed on an HPC cluster using one node, one task, and 16 CPUs per task as assigned by the SLURM scheduler (Yoo *et al.* 2003). As expected, the runtimes grew approximately following an O(N^2^) order with respect to the number of SNPs (N) (Table 8). Runtimes grew linearly, O(M), with respect to the number of individuals (M). Based on these runtimes, analysing ∼1 000 000 SNPs is feasible. In cases where analysis time becomes prohibitive, non-exhaustive algorithms might provide faster runtimes (Ponte-Fernández *et al.* 2022).

**Table 8:** PLINK epistasis runtimes as measured on HPC (one node, one task, 16 CPUs per task) for simulated datasets with varying numbers of SNPs (1000, 10 000, 100 000) and individuals (1000, 5000, 10 000).^a^

# of SNPs	# of individuals	Runtime
1000	1000	0.482 s
10 000	1000	6.76 s
100 000	1000	7.75 min
10 000	5000	22.2 s
10 000	10 000	43.1 s
**1** **000** **000**	**10** **000**	**129 h**

a The estimated values are bolded and underlined.

### 3.7 ABCD dataset

Of the seven methods used to analyse ABCD data, only four provided *P-*value: PLINK Epistasis, PLINK Boost, Matrix Epistasis, and REMMA. The suggestive significance cutoff of 1.64e−9 was applied to the results. Using this cutoff, PLINK Epistasis reported 14 suggestively significant results, PLINK BOOST reported 178, Matrix Epistasis reported 26, and REMMA reported 2. For MIDESP, the top 0.001% of results (which contained 180 SNP pairs) was examined. For MDR and QMDR, the top 500 results were reviewed. The interacting SNPs reported by PLINK Epistasis contained rs74991942, which maps onto the *DRD4* gene, while the interacting SNPs from PLINK BOOST included rs7131056, which maps onto the *DRD2* gene. None of the other reported SNPs from any tool mapped to genes previously associated with externalizing behavior.

The approximate runtimes for each tool are shown in Table 9. These runtimes are provided as examples, not for direct comparison, as different hardware was used for each tool. Furthermore, REMMA can be easily parallelized to run on multiple machines using its built-in functionality, and Matrix Epistasis can also be parallelized, according to the authors (Zhu and Fang 2018). MIDESP can be executed with more threads than we used in this study.

**Table 9:** Approximate runtimes for epistasis analysis of ABCD dataset consisting of 11 666 individuals and 24 699 SNPs.

Tool	Approximate runtime	Setup
PLINK Epistasis	5 min	High performance cluster (one node, one task, 16 CPUs per task). The CPUs present on HPC are Intel Quad Core E5-2450, E5-2650, E5530 XEON-EMT, E5520 XEON-EMT, and E5430 XEON-EMT. Plink uses up to 15 threads by default. MIDESP uses eight threads by default. REMMA execution was explicitly subdivided into 16 individual parallel components
REMMA	120 min	
PLINK Boost	180 min	
MIDESP	510 min	
MDR	480 min	Single computer with Intel i7-9700K CPU @ 3.60 GHz, eight cores, up to eight threads
Matrix Epistasis	480 min	
QMDR	660 min	

### 3.8 Overall performance

No tool excelled across all interaction types and metrics. However, some demonstrated better overall performance and utility than others. REMMA exhibited the highest overall sensitivity (55%), with many of its true positive detections ranked near the top, especially for dominant interactions, and it is capable of directly accounting for population stratification and relatedness. The number of false positives produced by REMMA was comparable to that of its competitors, as evidenced by *F1* scores in Table 5. PLINK Epistasis was another strong contender with an overall sensitivity of 49%. PLINK’s main advantage is fast runtime, and it is also one of the best supported tools with a long history of consistent updates. Matrix Epistasis performed identically to PLINK across all metrics, except that its runtime was slower. QMDR and MIDESP are viable alternatives to the linear models, although their overall performance was lower compared to REMMA, PLINK, and Matrix Epistasis.

On the case-control side, MDR is notable for its high overall sensitivity of 53% (using binarization threshold #1). However, its performance is highly dependent the choice of binarization threshold, as reflected in the differences observed across the two threshold sets. The utility of case-control tools largely depends on how easily a quantitative phenotype can be transformed into a case-control phenotype. BOOST appeared less sensitive to binarization threshold selection and exhibited the lowest false positive rate among all tools, but it also suffered from low overall sensitivity of 22%.

EpiSNP was the weakest performer of all examined tools. It failed to analyse the ABCD data, has difficult-to-use interface, and showed the lowest overall sensitivity of 15%. However, it exhibited the highest detection rate for recessive interactions, suggesting that it may still be useful as part of a multi-tool epistasis pipeline.

## 4 Discussion

We conducted a survey of the epistasis detection methods for quantitative phenotypes that are suitable for large scale pairwise epistasis scans. We further tested whether discretization could provide a practical alternative under some conditions. Six quantitative (EpiSNP, Matrix Epistasis, MIDESP, PLINK Epistasis, QMDR, and REMMA) and two case-control (BOOST and MDR) methods were selected for further evaluation in this study, representing five major models for detecting epistasis: GLMs, linear mixed models, linear regression, MI, and multifactor dimensionality reduction. The quantitative methods were used directly with the simulated quantitative phenotype datasets. The case-control methods were evaluated using discretized versions of the simulated datasets. The tools we selected are designed to detect second-order (pairwise) SNP–SNP interactions. There are some recently developed tools that did not make it into the study, but are worth mentioning such as: Epi-MEIF and QuadKAST (Saha *et al.* 2022, Fu *et al.* 2024). Epi-MEIF is primarily meant for higher-order epistasis analysis, which is beyond the scope of this publication. QuadKAST does not test individual SNP–SNP interactions one by one. Instead, as a kernel-based model, it tests the aggregate effect of all pairwise interactions within a set of SNPs. Both tools are well documented and readily accessible online. While this study is not exhaustive, our focus was on providing practical recommendations to the research community, prioritizing usability, and optimization over theoretical completeness (see Table 10).

**Table 10:** Summary of the pros and cons of each epistasis detection tool included in the study.

	Pros	Cons
EpiSNP	Best performance with recessive datasets Supports covariates	Worst overall performance Relatively slow runtime Difficult to use interface Has not been updated in >10 years May not be able to handle >5000 SNPs
Matrix	Good overall performance, especially with dominant datasets Supports covariates	Does not have an in-built support for parallel execution Has not been updated in >5 years
MIDESP	Good performance with multiplicative and XOR datasets Multithreading supported Supports discrete and continuous covariates Easy to use command line interface Updated within the last 5 years	Poor performance with dominant and especially recessive datasets
PLINK	Good overall performance, especially with dominant datasets Very fast runtime Easy to use command line interface with extensive documentation Actively maintained by a development team	Does not support covariates
QMDR	Good performance with XOR datasets Some covariate support Has graphical and command line interfaces	Middling overall performance Long runtime
REMMA	Good overall performance, especially with dominant datasets In-built support for parallel execution Can directly account for covariates, population structure, and family structures	Does not seem to be actively supported
BOOST	Low false positive rate with high average true positive ranking Implemented within a well-documented and actively supported tool (PLINK)	Low true positive rate Does not support covariates
MDR	Relatively good performance across all interaction types Some covariate support Has graphical and command line interfaces	The performance is highly dependent on choice of binarization threshold Long runtime

Although the selected tools were highly optimized, processing datasets with millions of SNPs and tens of thousands of individuals remain computationally demanding. Moreover, testing trillions of pairwise interactions can quickly diminish statistical power due to the large number of multiple comparisons that must be corrected. Therefore, a filtering step may be essential to improve efficiency and maintain power. It has been shown that, despite the vast number of potential interacting gene pairs, the actual genetic interaction density is relatively low (Schuldiner *et al.* 2005), and not all statistical interactions are biologically meaningful. To address this challenge, an exhaustive search method may be combined with a two-stage approach: first, a subset of genetic variants is selected in a filtering step, and then interactions are only tested among the variants within that subset. The filtering process may rely on statistical tests or biological knowledge. For instance, only genetic variants with significant main effects are subjected to interaction tests (Manning *et al.* 2009). Alternatively, individual interactions could be evaluated only among variants showing significant marginal epistasis effects, which refers to the combined pairwise interaction effects between a given variant and all others (Crawford *et al.* 2017). The filtering stage can also incorporate biological knowledge, such as pathways linked to diseases, protein–protein interaction networks, expression quantitative trait loci (eQTL) networks, and other regulatory networks. Statistical filtering has the advantage of being discovery-driven and mostly unbiased, whereas biological filtering is hypothesis-driven, making the findings more interpretable. We have successfully used the latter strategy, coupled with a bi-clustering algorithm, to identify interacting genes associated with alcohol use disorders in an admixed population using REMMA (Listopad and Peng 2026).

In this study, we evaluated the performance of epistasis detection methods using simulated datasets, but we recognize that real-life datasets pose additional challenges. In real-life applications, factors such as covariates, population structure, and family structure must be accounted for (Balvert *et al.* 2024). Notably, of all the tools analysed, only a few allow the inclusion of covariates directly into the epistasis analysis. Moreover, real-life datasets may contain multiple types of interactions simultaneously, whereas our simulation data focused on a specific type of interaction for each dataset.

The application of these tools to the ABCD data highlighted their advantages and limitations when handling real-world data. Tools that provide *P-*value for each SNP pair simplify the process of establishing significance. In contrast, other tools require additional, and sometimes time consuming steps such as permutation testing to establish significance (De *et al.* 2015). Real datasets also tend to be much larger than our simulated ones, with many containing millions of SNPs and an increasing number of individuals. Among the tools in this study, PLINK Epistasis and REMMA are the only ones that can be easily applied to large datasets with millions of SNPs and ten thousand individuals. While REMMA is slower than PLINK, it offers the advantage of being highly parallelizable. When analysing the ABCD data, only PLINK Epistasis and BOOST yielded the results that mapped to genes previously associated with externalizing behavior. These results should be interpreted with caution, as they are consistent with previous findings but do not constitute robust validation. Moreover, these results were obtained using a suggestive significance cutoff, and most of the reported interactions did not map onto any genes previously associated with externalizing behavior. Other important considerations in analysis of real data are population stratification, individual relatedness, and other covariates such as sex and age. REMMA stands out in this regard, as it can directly account for all of these factors. While some other tools can handle covariates, they may not fully account for complex population structures or individual relatedness directly.

Our results demonstrate that identifying interacting SNP pairs is challenging due to the large number of false positives reported by each detection method. One possible reason for the large false positive rate is the presence of realistic linkage disequilibrium structure within the EpiGEN simulated data. In a 2018 study, Chatelain *et al.* demonstrated that some epistasis detection methods generate inflated false positive rates given LD between causal SNPs (Chatelain *et al.* 2018). Given that all simulated datasets were generated using chromosome 1 of the CEU HapMap3 cohort the causal SNPs could be in LD in some of the datasets. While true positives are generally found toward the top of the sorted hit lists, this is not always the case. Consequently, further validation, including computational and biological validation, is necessary when interpreting results from epistasis detection analysis. Additionally, it is important to remember that even true statistical epistasis interactions may not always translate into meaningful biological interactions.

An unexpected finding of our study was that case-control methods can perform relatively well with discretized version of the quantitative phenotype. In particular, MDR, demonstrated good performance with the discretized data, despite the loss of information inherent in converting a quantitative phenotype into a case-control phenotype. However, MDR’s performance was highly dependent on the choice of the binarization threshold. When presented with quantitative phenotype that can be easily converted into a case-control one, using case-control epistasis detection methods with such discretized data may be a reasonable approach.

Our study has demonstrated that existing epistasis detection methods generally provide promising results for quantitative traits. There is however a lack of consistency in the findings across various models, which is not surprising given that the modeling frameworks differ in their respective abilities to detect specific types of epistasis. Based on our analysis, we recommend using a consensus of multiple methods to identify interacting SNP pairs in datasets with quantitative phenotypes (D’Silva *et al.* 2022). In this approach, significant interactions detected by majority of several distinct methods are considered for further replication. This is preferable to relying on a single tool, as we cannot predict which types of epistasis may be present in real data samples a priori, and none of the methods evaluated in this study excelled at detecting all interaction types. However, if a single tool is preferred for convenience, PLINK Epistasis and REMMA are the most readily applicable. PLINK is best suited for datasets with low or no individual relatedness, while REMMA is uniquely suited for datasets with complex population structures and high level of individual relatedness. Notably, discretizing a quantitative phenotype (when applicable) followed by applying case-control epistasis detection methods is a viable approach.

## Key conclusions and recommendations

- No single tool excels at detecting all types of epistasis for quantitative phenotypes.
- Matrix Epistasis, PLINK Epistasis, and REMMA are best at detecting dominant interactions.
- MDR and MIDESP perform best at identifying multiplicative and XOR interactions.
- EpiSNP excels at detecting recessive interactions.
- PLINK Epistasis and REMMA are well suited for and readily applicable to real-world data.
- Combining tools may be the most effective approach, as the interaction types are typically unknown in real datasets.

## Supplementary Material

btag344_Supplementary_Data

## Data Availability

All of the EpiGEN simulated data and code used to process it can be found in the following GitHub repository: https://github.com/staslist/Epistasis_Review.
