# Supplementary material for: Evaluation of epistasis detection methods for quantitative phenotypes
Source: Bioinformatics. 2026 Jun 4;42(6):btag344. doi: 10.1093/bioinformatics/btag344 (PMC13264488; doi:10.1093/bioinformatics/btag344)
Supplement: btag344_Supplementary_Data [file btag344_supplementary_data.zip › SupplementalFile_S3.docx]

Epistasis Tool Comparison

Compared epistasis detection tools for quantitative phenotype.

Currently the tools are: Plink’s Epistasis, Plink’s Boost (Discretized Phenotype), Matrix Epistasis, MIDESP, MDR (Discretized Phenotype), QMDR, EpiSNP, REMMA.

A report with results and brief discussion was generated for each tool.

The goal of this document is to compare the performance of all the included tools.

Contents

[SECTION 1 EPISTASIS TYPES, BIOLOGICAL PLAUSIBILITY 2](#_Toc222959644)

[SECTION 2 TRUE POSITIVES DETECTED 3](#_Toc222959645)

[SECTION 3 TRUE POSITIVE AVERAGE POSITION 5](#_Toc222959646)

[SECTION 4 F1 SCORE 9](#_Toc222959647)

[SECTION 5 MULTIPLICATIVE PURITY, INTERACTION ALPHA IMPACT 11](#_Toc222959648)

[SECTION 6 REGULAR DOMINANT PURITY, INTERACTION ALPHA IMPACT 13](#_Toc222959649)

[SECTION 7 RECESSIVE PURITY, INTERACTION ALPHA IMPACT 15](#_Toc222959650)

[SECTION 8 XOR PURITY, INTERACTION ALPHA IMPACT 17](#_Toc222959651)

[SECTION 9 LARGE DOMINANT DATASETS - IMPACT OF MAF, NUMBER OF SNPS, NUMBER OF INDIVIDUALS 19](#_Toc222959652)

[SECTION 10 TRUE POSITIVE RATE BY INTERACTION ALPHA AND PURITY 22](#_Toc222959653)

[SECTION 11 ASSEMBLY OF TOOLS 25](#_Toc222959654)

# SECTION 1 EPISTASIS TYPES, BIOLOGICAL PLAUSIBILITY

We used EPIGEN to generate following interaction types:

- Multiplicative
- Dominant
- Recessive
- XOR

These epistasis interaction types are arranged in order of presumable (hypothesized) difficulty of detection.


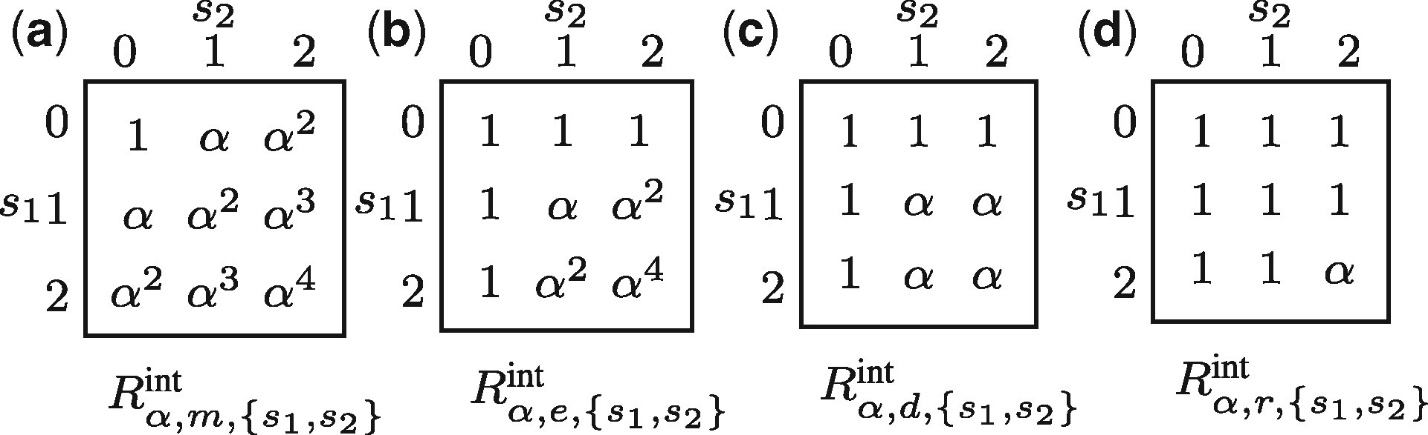


Interaction Types: multiplicative (m), exponential (e), dominant (d), recessive (r). The x and y axis are genotypes of snp1 and snp2. The genotype value indicates number of minor alleles (0, 1, or 2).

Additionally, we implemented XOR interaction defined as follows: if one SNP genotype is in [1, 2] and the other SNP genotype is in [0] interaction alpha = alpha, otherwise interaction alpha = 1.

Total number of Epigen Datasets: 46; 20 multiplicative datasets, 8 recessive datasets, 8 dominant datasets, and 4 XOR datasets.

Total number of interacting pairs across all datasets: 112; 56 multiplicative pairs, 32 XOR pairs, 12 recessive pairs, 12 dominant pairs.

Biological Plausibility:

Dominant / Recessive (EPIGEN definition): YES - several examples are provided in the Nature Education Scitable page on Epistasis (Miko, I. (2008) Epistasis: Gene interaction and phenotype effects. *Nature Education* 1(1):197) NOTE: the definition of dominant / recessive epistasis is different within the citation from that of EPIGEN. An example of EPIGEN dominant interaction could be the one with ratio of 9:7. While an example of EPIGEN recessive interaction could be the one with ratio of 15:1.

XOR: unknown, I could not find any clear-cut examples of these interactions.

Multiplicative: unclear, generally the meaning of multiplicative epistasis within the community is different from the definition used within EpiGEN.

# SECTION 2 TRUE POSITIVES DETECTED

Pairs detected by tool:

- EpiSNP: 21/144 (15%)
  - Multiplicative: 0/56 (0%)
  - Regular Dominant: 0/12 (0%)
  - Recessive: 8/12 (67%)
  - XOR: 2/32 (6%)
  - Large Dominant: 11/32 (34%)
- Matrix Epistasis: 70/144 (49%)
  - Multiplicative: 7/56 (12%)
  - Regular Dominant: 12/12 (100%)
  - Recessive: 6/12 (50%)
  - XOR: 13/32 (41%)
  - Large Dominant: 32/32 (100%)
- MDR (Discretized Threshold #1): 77/144 (53%)
  - Multiplicative: Multiplicative 28/56 (50%)
  - Regular Dominant: 7/12 (58%)
  - Recessive: 5/12 (42%)
  - XOR: 23/32 (72%)
  - Large Dominant: 14/32(44%)
- MDR (Discretized Threshold #2): 64/144 (44%)
  - Multiplicative: 36/56 (64%)
  - Regular Dominant: 4/12 (33%)
  - Recessive: 3/12 (25%)
  - XOR: 21/32 (66%)
  - Large Dominant: 0/32 (0%)
- MIDESP: 48/144 (33%)
  - Multiplicative: 25/56 (45%)
  - Regular Dominant: 4/12 (33%)
  - Recessive: 0/12 (0%)
  - XOR: 14/32 (44%)
  - Large Dominant: 5/32 (16%)
- Plink BOOST (Discretized Threshold #1): 31/144 (22%)
  - Multiplicative: 2/56 (4%)
  - Regular Dominant: 2/12 (17%)
  - Recessive: 0 (0%)
  - XOR: 5/32 (16%)
  - Large Dominant: 22/32 (69%)
- Plink BOOST (Discretized Threshold #2): 32/144 (22%)
  - Multiplicative: 1/56 (2%)
  - Regular Dominant: 3/12 (25%)
  - Recessive: 0/12 (0%)
  - XOR: 6/32 (19%)
  - Large Dominant: 22/32 (69%)
- Plink Epistasis: 70/144 (49%)
  - Multiplicative: 7/56 (12%)
  - Regular Dominant: 12/12 (100%)
  - Recessive: 6/12 (50%)
  - XOR: 13/32 (41%)
  - Large Dominant: 32/32 (100%)
- QMDR: 47/144 (33%)
  - Multiplicative: 16/56 (29%)
  - Regular Dominant: 7/12 (58%)
  - Recessive: 0/12 (0%)
  - XOR: 19/32 (59%)
  - Large Dominant: 5/32 (16%)
- REMMA: 79/144 (55%)
  - Multiplicative: 9/56 (16%)
  - Regular Dominant: 12/12 (100%)
  - Recessive: 6/12 (50%)
  - XOR: 20/32 (62%)
  - Large Dominant: 32/32 (100%)

# SECTION 3 TRUE POSITIVE AVERAGE POSITION

Now let us examine average true positive position by interaction per tool. Note that in computing the average we only account for datasets wherein at least one pair was correctly detected. Also note that these are penalized average positions, that is when a given dataset had > 1 interacting pairs and not all pairs were detected, the undetected pairs were considered to be at the “end” of the sorted list.

The detected pairs were sorted by p-value in Plink Epistasis, Matrix Epistasis, EpiSNP, and REMMA, by mutual information in MIDESP, by accuracy in MDR, and by t-statistic in QMDR.

- EpiSNP: 556.88
  - Multiplicative: NA
  - Regular Dominant: NA
  - Recessive: 500.92
  - XOR: 147.19
  - Large Dominant: 749.42
- Matrix Epistasis: 161.03
  - Multiplicative: 90.29
  - Regular Dominant: 1.25
  - Recessive: 414.00
  - XOR: 52.03
  - Large Dominant: 204.25
- MDR (Discretized Threshold #1): 166.10
  - Multiplicative: 115.59
  - Regular Dominant: 153.57
  - Recessive: 230.50
  - XOR: 169.56
  - Large Dominant: 212.75
- MDR (Discretized Threshold #2): 130.13
  - Multiplicative: 89.40
  - Regular Dominant: 183.00
  - Recessive: 215.50
  - XOR: 196.53
  - Large Dominant: NA
- MIDESP: 661.61
  - Multiplicative: 53.83
  - Regular Dominant: 624.50
  - Recessive: NA
  - XOR: 126.22
  - Large Dominant: 4856.67
- Plink BOOST (Discretized Threshold #1): 1.73
  - Multiplicative: 1.00
  - Regular Dominant: 1.50
  - Recessive: NA
  - XOR: 3.19
  - Large Dominant: 1.64
- Plink BOOST (Discretized Threshold #2): 1.85
  - Multiplicative: 1.0
  - Regular Dominant: 1.33
  - Recessive: NA
  - XOR: 3.44
  - Large Dominant: 1.85
- Plink Epistasis: 162.60
  - Multiplicative: 91.93
  - Regular Dominant: 1.25
  - Recessive: 421.83
  - XOR: 53.94
  - Large Dominant: 204.16
- QMDR: 175.51
  - Multiplicative: 211.78
  - Regular Dominant: 42.92
  - Recessive: NA
  - XOR: 250.22
  - Large Dominant: 190.88
- REMMA: 333.71
  - Multiplicative: 336.02
  - Regular Dominant: 1.62
  - Recessive: 604.33
  - XOR: 204.34
  - Large Dominant: 429.47

Lets also examine what the average true positive positions looks like without the penalty mentioned above. Once again, we only include datasets wherein at least a single pair was detected. Let us also list the individual true positive positions (when at least one pair was detected in a dataset).

Values are listed as follows: mean; median – individual dataset average true positive positions.

- EpiSNP:
  - Multiplicative: NA
  - Regular Dominant: NA
  - Recessive: 97.25; 44.00 - [8, 19, 27, 61, 126.5, 342]
  - XOR: 15.50; 15.50 - [6, 25]
  - Large Dominant: 671.58; 651.75 - [199.5, 391.5, 482, 821.5, 983.5, 1151.5]
- Matrix Epistasis:
  - Multiplicative: 6.00; 1.00 - [1, 1, 1, 1, 3, 4, 31]
  - Regular Dominant: 1.25; 1.25 - [1, 1, 1, 1, 1.5, 1.5, 1.5, 1.5]
  - Recessive: 91.67; 78.50 - [9, 43, 57, 100, 149, 192]
  - XOR: 26.90; 20.50 - [4, 8, 33, 62.6]
  - Large Dominant: 204.25; 31.50 - [1.5, 1.5, 1.5, 2, 2, 4.5, 10, 31, 32, 50, 76, 146.5, 351, 492.5, 1027.5, 1038.5]
- MDR (Discretized Threshold #1):
  - Multiplicative: 12.36; 3.25 - [1, 1, 1, 1, 1, 1, 2, 3, 3.5, 4, 7.5, 8.5, 25, 42.5, 43, 52.75]
  - Regular Dominant: 91.86; 1.00 - [1, 1, 1, 1, 7, 181, 451]
  - Recessive: 120.20; 70.00 - [1, 9, 70, 131, 390]
  - XOR: 40.33; 38.26 - [23.14, 37.66, 38.864, 61.67]
  - Large Dominant: 7.29; 5.00 - [3, 3, 3, 3.5, 4, 5, 5, 5, 5, 12, 13, 26]
- MDR (Discretized Threshold #2):
  - Multiplicative: 15.99; 3.75 - [1, 1, 1, 1, 1, 2, 2, 3, 3.5, 4, 4, 4.5, 5.5, 10, 47, 50.6, 68.8, 78]
  - Regular Dominant: 115.00; 4.00 - [1, 1, 7, 451]
  - Recessive: 197.00; 131.00 - [70, 131, 390]
  - XOR: 37.53; 37.79 - [23.2, 25.83, 49.75, 51.33]
  - Large Dominant: NA
- MIDESP:
  - Multiplicative: 7.57; 3.00 - [1, 1, 1, 1, 1, 1, 2, 2, 3, 3, 3, 3, 7, 10, 13.5, 20.5, 55.67]
  - Regular Dominant: 624.50; 1.00 - [1, 1, 1871.5]
  - Recessive: NA
  - XOR: 12.02; 8.33 - [1, 5.67, 11, 30.4]
  - Large Dominant: 4719.33; 4996.50 - [4154.5, 4996.5, 5007]
- Plink BOOST (Discretized Threshold #1):
  - Multiplicative: 1.00; 1.00 - [1, 1]
  - Regular Dominant: 1.00; 1.00 - [1, 1]
  - Recessive: NA
  - XOR: 2.00; 2.00 - [1, 3]
  - Large Dominant: 1.64; 1.50 - [1.5, 1.5, 1.5, 1.5, 1.5, 1.5, 1.5, 1.5, 2, 2, 2]
- Plink BOOST (Discretized Threshold #2):
  - Multiplicative: 1.00; 1.00 - [1]
  - Regular Dominant: 1.00; 1.00 - [1, 1, 1]
  - Recessive: NA
  - XOR: 2.00; 2.00 - [1.5, 2.5]
  - Large Dominant: 1.71; 1.50 - [1, 1, 1.5, 1.5, 1.5, 1.5, 1.5, 1.5, 1.5, 2, 2.5, 3.5]
- Plink Epistasis:
  - Multiplicative: 5.86; 1.00 - [1, 1, 1, 1, 3, 4, 30]
  - Regular Dominant: 1.25; 1.25 - [1, 1, 1, 1, 1.5, 1.5, 1.5, 1.5]
  - Recessive: 91.33; 78.50 - [9, 42, 57, 100, 149, 191]
  - XOR: 26.90; 20.50 - [4, 8, 33, 62.6]
  - Large Dominant: 204.16; 31.50 - [1.5, 1.5, 1.5, 2, 2, 4.5, 10, 31, 32, 50, 76, 146.5, 351, 492.5, 1026, 1038.5]
- QMDR:
  - Multiplicative: 102.39; 24.83 - [1, 2, 2, 3, 3, 5, 44.67, 138, 147, 186, 296, 401]
  - Regular Dominant: 1.25; 1.00 - [1, 1, 1, 1, 1.5, 2]
  - Recessive: NA
  - XOR: 93.26; 101.80 - [27.28, 61.6, 142, 142.17]
  - Large Dominant: 3.50; 1.50 - [1, 1, 2, 10]
- REMMA:
  - Multiplicative: 128.12; 16.50 - [1, 2, 2, 15, 18, 38, 305, 644]
  - Regular Dominant: 1.62; 1.50 - [1, 1, 1, 1, 2, 2, 2, 3]
  - Recessive: 154.50; 131.50 - [11, 38, 77, 186, 256, 359]
  - XOR: 76.25; 31.42 - [6, 17.33, 45.5, 236.17]
  - Large Dominant: 429.47; 8.50 - [1.5, 1.5, 2, 2, 2, 2, 2.5, 8.5, 8.5, 77, 90.5, 174, 416, 969, 1570, 3544.5]

As we can see true positives that are detected, are often the highest ranked hit. However, many are also found outside of top 100.

# SECTION 4 F1 SCORE

F1 score ranges from 0 to 1 with 1 indicating perfect performance (no false positives or false negatives).

- EpiSNP:
  - Multiplicative: 0.00000
  - Regular Dominant: 0.00000
  - Recessive: 0.00110
  - XOR: 0.02653
  - Large Dominant: 0.00052
- Matrix Epistasis:
  - Multiplicative: 0.01715
  - Regular Dominant: 0.01017
  - Recessive: 0.00148
  - XOR: 0.14969
  - Large Dominant: 0.00417
- MDR (Discretized Threshold #1):
  - Multiplicative: 0.00555
  - Regular Dominant: 0.00349
  - Recessive: 0.00249
  - XOR: 0.02264
  - Large Dominant: 0.00349
- MDR (Discretized Threshold #2):
  - Multiplicative: 0.00713
  - Regular Dominant: 0.00199
  - Recessive: 0.00150
  - XOR: 0.02067
  - Large Dominant: 0.00000
- MIDESP:
  - Multiplicative: 0.03286
  - Regular Dominant: 0.00136
  - Recessive: 0.00
  - XOR: 0.04491
  - Large Dominant: 0.00008
- Plink BOOST (Discretized Threshold #1):
  - Multiplicative: 0.10000
  - Regular Dominant: 0.16667
  - Recessive: 0
  - XOR: 0.20940
  - Large Dominant: 0.25298
- Plink BOOST (Discretized Threshold #2):
  - Multiplicative: 0.05000
  - Regular Dominant: 0.29167
  - Recessive: 0
  - XOR: 0.25385
  - Large Dominant: 0.43201
- Plink Epistasis:
  - Multiplicative: 0.01423
  - Regular Dominant: 0.00995
  - Recessive: 0.00145
  - XOR: 0.14140
  - Large Dominant: 0.00410
- QMDR:
  - Multiplicative: 0.00317
  - Regular Dominant: 0.00349
  - Recessive: 0.00
  - XOR: 0.01870
  - Large Dominant: 0.00125
- REMMA:
  - Multiplicative: 0.00241
  - Regular Dominant: 0.00501
  - Recessive: 0.00103
  - XOR: 0.12694
  - Large Dominant: 0.00165

# SECTION 5 MULTIPLICATIVE PURITY, INTERACTION ALPHA IMPACT

The multiplicative datasets provide a good way to examine the impact of purity and interaction alpha on rate of detection.

All average true positive positions in this section are penalized. Only multiplicative datasets are considered. Zero indicates no true positives were detected. After => is total number of true positives detected for that category divided by total number of interacting pairs in that category.

- EpiSNP:
  - Interaction Alpha 1.25: [0, 0, 0, 0, 0, 0]=>0/22
  - Interaction Alpha 1.5: [0, 0, 0, 0, 0, 0]=>0/22
  - Interaction Alpha 2: [0, 0, 0, 0]=>0/6
  - Interaction Alpha 3: [0, 0, 0, 0]=>0/6
  - Pure: [0, 0, 0, 0, 0, 0, 0, 0, 0, 0]=>0/28
  - Impure: [0, 0, 0, 0, 0, 0, 0, 0, 0, 0]=>0/28
- Matrix Epistasis:
  - Interaction Alpha 1.25: [0, 0, 0, 0, 0, 0]=>0/22
  - Interaction Alpha 1.5: [0, 0, 0, 0, 0, 6.5]=>1/22
  - Interaction Alpha 2: [0, 1, 59.5, 290.5]=>3/6
  - Interaction Alpha 3: [0, 1, 1, 272.5]=>3/6
  - Pure: [0, 0, 0, 0, 0, 1, 1, 6.5, 59.5, 272.5]=>5/28
  - Impure: [0, 0, 0, 0, 0, 0, 0, 0, 1, 290.5]=>2/28
- MDR (discretized threshold #1):
  - Interaction Alpha 1.25: [0, 0, 1, 3.5, 322.5, 386.375]=>8/22
  - Interaction Alpha 1.5: [0, 0, 1, 4, 276.875, 329.25]=>10/22
  - Interaction Alpha 2: [1, 2, 8.5, 251]=>5/6
  - Interaction Alpha 3: [1, 3, 7.5, 251]=>5/6
  - Pure: [1, 1, 1, 1, 3.5, 4, 7.5, 251, 322.5, 329.25]=>17/28
  - Impure: [0, 0, 0, 0, 2, 3, 8.5, 251, 276.875, 386.375]=>11/28
- MDR (discretized threshold #2):
  - Interaction Alpha 1.25: [0, 0, 1, 3.5, 230.875, 342.375]=>11/22
  - Interaction Alpha 1.5: [1, 3, 4, 10, 219.5, 274]=>15/22
  - Interaction Alpha 2: [1, 4, 5.5, 251.5]=>5/6
  - Interaction Alpha 3: [1, 1, 4.5, 251.5]=>5/6
  - Pure: [1, 1, 1, 1, 3.5, 4, 4.5, 219.5, 230.875, 251.5]=>21/28
  - Impure: [0, 0, 1, 3, 4, 5.5, 10, 251.5, 274, 342.375]=>15/28
- MIDESP:
  - Interaction Alpha 1.25: [0, 0, 1, 70.5, 115, 152.625]=>5/22
  - Interaction Alpha 1.5: [0, 1, 2, 3, 99.75, 385.25]=>11/22
  - Interaction Alpha 2: [1, 3, 11.5, 33.5]=>4/6
  - Interaction Alpha 3: [1, 1, 3, 31]=>5/6
  - Pure: [1, 1, 1, 1, 3, 3, 11.5, 70.5, 115, 385.25]=>14/28
  - Impure: [0, 0, 0, 1, 2, 3, 31, 33.5, 99.75, 152.625]=>11/28
- Plink BOOST (discretized threshold #1):
  - Interaction Alpha 1.25: [0, 0, 0, 0, 0, 1]=>1/22
  - Interaction Alpha 1.5: [0, 0, 0, 0, 0, 1]=>1/22
  - Interaction Alpha 2: [0, 0, 0, 0]=>0/6
  - Interaction Alpha 3: [0, 0, 0, 0]=>0/6
  - Pure: [0, 0, 0, 0, 0, 0, 0, 0, 0, 0]=>0/28
  - Impure: [0, 0, 0, 0, 0, 0, 0, 0, 1, 1]=>2/28
- Plink BOOST (discretized threshold #2):
  - Interaction Alpha 1.25: [0, 0, 0, 0, 0, 1]=>1/22
  - Interaction Alpha 1.5: [0, 0, 0, 0, 0, 0]=>0/22
  - Interaction Alpha 2: [0, 0, 0, 0]=>0/6
  - Interaction Alpha 3: [0, 0, 0, 0]=>0/6
  - Pure: [0, 0, 0, 0, 0, 0, 0, 0, 0, 0]=>0/28
  - Impure: [0, 0, 0, 0, 0, 0, 0, 0, 0, 1]=>1/28
- Plink Epistasis:
  - Interaction Alpha 1.25: [0, 0, 0, 0, 0, 0]=>0/22
  - Interaction Alpha 1.5: [0, 0, 0, 0, 0, 7.5]=>1/22
  - Interaction Alpha 2: [0, 1, 61, 293.5]=>3/6
  - Interaction Alpha 3: [0, 1, 1, 278.5]=>3/6
  - Pure: [0, 0, 0, 0, 0, 1, 1, 7.5, 61, 278.5]=>5/28
  - Impure: [0, 0, 0, 0, 0, 0, 0, 0, 1, 293.5]=>2/28
- QMDR:
  - Interaction Alpha 1.25: [0, 0, 147, 251.5, 329.875, 475.375]=>6/22
  - Interaction Alpha 1.5: [0, 0, 3, 138, 461.625, 476]=>6/22
  - Interaction Alpha 2: [0, 0, 1, 253]=>2/6
  - Interaction Alpha 3: [0, 0, 2, 3]=>2/6
  - Pure: [0, 1, 3, 3, 138, 147, 251.5, 253, 329.875, 476]=>13/28
  - Impure: [0, 0, 0, 0, 0, 0, 0, 2, 461.625, 475.375]=>3/28
- REMMA:
  - Interaction Alpha 1.25: [0, 0, 0, 0, 0, 140.5]=>1/22
  - Interaction Alpha 1.5: [0, 0, 0, 0, 0, 720.125]=>1/22
  - Interaction Alpha 2: [0, 15, 305, 883]=>4/6
  - Interaction Alpha 3: [0, 1, 2, 621.5]=>3/6
  - Pure: [0, 0, 0, 0, 0, 1, 15, 305, 621.5, 720.125]=>6/28
  - Impure: [0, 0, 0, 0, 0, 0, 0, 2, 140.5, 883]=>3/28

Across all tested tools that had significant rates of detection multiplicative interactions with larger alpha and pure status were more successfully detected.

# SECTION 6 REGULAR DOMINANT PURITY, INTERACTION ALPHA IMPACT

All average true positive positions in this section are penalized. Only dominant datasets are considered. Zero indicates no true positives were detected.

- EpiSNP:
  - Interaction Alpha 8: [0, 0, 0, 0]=>0/6
  - Interaction Alpha 16: [0, 0, 0, 0]=>0/6
  - Pure: [0, 0, 0, 0]=>0/6
  - Impure: [0, 0, 0, 0]=>0/6
- Matrix Epistasis:
  - Interaction Alpha 8: [1, 1, 1.5, 1.5]=>6/6
  - Interaction Alpha 16: [1, 1, 1.5, 1.5]=>6/6
  - Pure: [1, 1, 1.5, 1.5]=>6/6
  - Impure: [1, 1, 1.5, 1.5]=>6/6
- MDR (discretized threshold #1):
  - Interaction Alpha 8: [0, 1, 1, 254]=>3/6
  - Interaction Alpha 16: [1, 1, 341, 476]=>4/6
  - Pure: [1, 1, 254, 476]=>4/6
  - Impure: [0, 1, 1, 341]=>3/6
- MDR (discretized threshold #2):
  - Interaction Alpha 8: [0, 0, 1, 254]=>2/6
  - Interaction Alpha 16: [0, 0, 1, 476]=>2/6
  - Pure: [1, 1, 254, 476]=>4/6
  - Impure: [0, 0, 0, 0]=>0/6
- MIDESP:
  - Interaction Alpha 8: [0, 0, 1, 1871.5]=>3/6
  - Interaction Alpha 16: [0, 0, 0, 1]=>1/6
  - Pure: [0, 0, 1, 1]=>2/6
  - Impure: [0, 0, 0, 1871.5]=>2/6
- Plink BOOST (discretized threshold #1):
  - Interaction Alpha 8: [0, 0, 0, 1.5]=>1/6
  - Interaction Alpha 16: [0, 0, 0, 1.5]=>1/6
  - Pure: [0, 0, 0, 1.5]=>1/6
  - Impure: [0, 0, 0, 1.5]=>1/6
- Plink BOOST (discretized threshold #2):
  - Interaction Alpha 8: [0, 0, 1.5, 1.5]=>2/6
  - Interaction Alpha 16: [0, 0, 0, 1]=>1/6
  - Pure: [0, 0, 0, 1.5]=>1/6
  - Impure: [0, 0, 1, 1.5]=>2/6
- Plink Epistasis:
  - Interaction Alpha 8: [1, 1, 1.5, 1.5]=>6/6
  - Interaction Alpha 16: [1, 1, 1.5, 1.5]=>6/6
  - Pure: [1, 1, 1.5, 1.5]=>6/6
  - Impure: [1, 1, 1.5, 1.5]=>6/6
- QMDR:
  - Interaction Alpha 8: [0, 1, 1, 1.5]=>4/6
  - Interaction Alpha 16: [0, 1, 2, 251]=>3/6
  - Pure: [1, 1, 1.5, 251]=>5/6
  - Impure: [0, 0, 1, 2]=>2/6
- REMMA:
  - Interaction Alpha 8: [1, 1, 2, 3]=>6/6
  - Interaction Alpha 16: [1, 1, 2, 2]=>6/6
  - Pure: [1, 1, 2, 2]=>6/6
  - Impure: [1, 1, 2, 3]=>6/6

# SECTION 7 RECESSIVE PURITY, INTERACTION ALPHA IMPACT

All average true positive positions in this section are penalized. Only recessive datasets are considered. Zero indicates no true positives were detected.

- EpiSNP:
  - Interaction Alpha 8: [0, 8, 342, 1493.5]=>4/6
  - Interaction Alpha 16: [0, 61, 126.5, 974.5]=>4/6
  - Pure: [8, 61, 126.5, 342]=>6/6
  - Impure: [0, 0, 974.5, 1493.5]=>2/6
- Matrix Epistasis:
  - Interaction Alpha 8: [0, 192, 333.5, 766]=>3/6
  - Interaction Alpha 16: [0, 149, 309, 734.5]=>3/6
  - Pure: [149, 192, 309, 333.5]=>4/6
  - Impure: [0, 0, 734.5, 766]=>2/6
- MDR (discretized threshold #1):
  - Interaction Alpha 8: [0, 0, 70, 255]=>2/6
  - Interaction Alpha 16: [0, 131, 251, 445.5]=>3/6
  - Pure: [0, 70, 131, 445.5]=>3/6
  - Impure: [0, 0, 251, 255]=>2/6
- MDR (discretized threshold #2):
  - Interaction Alpha 8: [0, 0, 0, 70]=>1/6
  - Interaction Alpha 16: [0, 0, 131, 445.5]=>2/6
  - Pure: [0, 70, 131, 445.5]=>3/6
  - Impure: [0, 0, 0, 0]=>0/6
- MIDESP:
  - Interaction Alpha 8: [0, 0, 0, 0]=>0/6
  - Interaction Alpha 16: [0, 0, 0, 0]=>0/6
  - Pure: [0, 0, 0, 0]=>0/6
  - Impure: [0, 0, 0, 0]=>0/6
- Plink BOOST (discretized threshold #1):
  - Interaction Alpha 8: [0, 0, 0, 0]=>0/6
  - Interaction Alpha 16: [0, 0, 0, 0]=>0/6
  - Pure: [0, 0, 0, 0]=>0/6
  - Impure: [0, 0, 0, 0]=>0/6
- Plink BOOST (discretized threshold #2):
  - Interaction Alpha 8: [0, 0, 0, 0]=>0/6
  - Interaction Alpha 16: [0, 0, 0, 0]=>0/6
  - Pure: [0, 0, 0, 0]=>0/6
  - Impure: [0, 0, 0, 0]=>0/6
- Plink Epistasis:
  - Interaction Alpha 8: [0, 191, 342, 780.5]=>3/6
  - Interaction Alpha 16: [0, 149, 315.5, 753]=>3/6
  - Pure: [149, 191, 315.5, 342]=>4/6
  - Impure: [0, 0, 753, 780.5]=>2/6
- QMDR:
  - Interaction Alpha 8: [0, 0, 0, 0]=>0/6
  - Interaction Alpha 16: [0, 0, 0, 0]=>0/6
  - Pure: [0, 0, 0, 0]=>0/6
  - Impure: [0, 0, 0, 0]=>0/6
- REMMA:
  - Interaction Alpha 8: [0, 359, 597.5, 924.5]=>3/6
  - Interaction Alpha 16: [0, 256, 414.5, 1074.5]=>3/6
  - Pure: [256, 359, 414.5, 597.5]=>4/6
  - Impure: [0, 0, 924.5, 1074.5]=>2/6

# SECTION 8 XOR PURITY, INTERACTION ALPHA IMPACT

All average true positive positions in this section are penalized. Only XOR datasets are considered. Zero indicates no true positives were detected.

- EpiSNP:
  - Interaction Alpha 1.5: [0, 12.125]=>1/16
  - Interaction Alpha 2: [0, 282.25]=>1/16
  - Pure: [0, 282.25]=>1/16
  - Impure: [0, 12.125]=>1/16
- Matrix Epistasis:
  - Interaction Alpha 1.5: [6.5, 59.25]=>6/16
  - Interaction Alpha 2: [41, 101.375]=>7/16
  - Pure: [59.25, 101.375]=>8/16
  - Impure: [6.5, 41]=>5/16
- MDR (discretized threshold #1):
  - Interaction Alpha 1.5: [96.625, 171.5]=>13/16
  - Interaction Alpha 2: [82.875, 327.25]=>10/16
  - Pure: [82.875, 96.625]=>14/16
  - Impure: [171.5, 327.25]=>9/16
- MDR (discretized threshold #2):
  - Interaction Alpha 1.5: [163.75, 275.375]=>10/16
  - Interaction Alpha 2: [144.625, 202.375]=>11/16
  - Pure: [144.625, 163.75]=>12/16
  - Impure: [202.375, 275.375]=>9/16
- MIDESP:
  - Interaction Alpha 1.5: [93.625, 137]=>7/16
  - Interaction Alpha 2: [31.75, 242.5]=>7/16
  - Pure: [31.75, 93.625]=>11/16
  - Impure: [137, 242.5]=>3/16
- Plink BOOST (discretized threshold #1):
  - Interaction Alpha 1.5: [0, 1.87]=>1/16
  - Interaction Alpha 2: [0, 4.5]=>4/16
  - Pure: [1.875, 4.5]=>5/16
  - Impure: [0, 0]=>0/16
- Plink BOOST (discretized threshold #2):
  - Interaction Alpha 1.5: [0, 0]=>0/16
  - Interaction Alpha 2: [2.625, 4.25]=>6/16
  - Pure: [0, 4.25]=>4/16
  - Impure: [0, 2.625]=>2/16
- Plink Epistasis:
  - Interaction Alpha 1.5: [7.125, 61.75]=>6/16
  - Interaction Alpha 2: [42.5, 104.375]=>7/16
  - Pure: [61.75, 104.375]=>8/16
  - Impure: [7.125, 42.5]=>5/16
- QMDR:
  - Interaction Alpha 1.5: [226.375, 231.875]=>11/16
  - Interaction Alpha 2: [86.5, 456.125]=>8/16
  - Pure: [86.5, 231.875]=>13/16
  - Impure: [226.375, 456.125]=>6/16
- REMMA:
  - Interaction Alpha 1.5: [12.375, 69.125]=>11/16
  - Interaction Alpha 2: [45.25, 690.625]=>9/16
  - Pure: [69.125, 690.625]=>12/16
  - Impure: [12.375, 45.25]=>8/16

# SECTION 9 LARGE DOMINANT DATASETS - IMPACT OF MAF, NUMBER OF SNPS, NUMBER OF INDIVIDUALS

All average true positive positions in this section are penalized. Only large dominant datasets are considered. Zero indicates no true positives were detected.

- EpiSNP:
  - SNP1000: [0, 949]=>1/4 (25%)
  - SNP2500: [0, 0, 0, 0, 0, 199.5, 391.5, 983.5]=>6/16 (37.5%)
  - SNP5000: [0, 0, 0, 0, 821.5, 1151.5]=>4/12 (33%)
  - IND1000: [0, 0]=>0/4 (0%)
  - IND2500: [0, 0, 0, 0, 199.5, 391.5, 949, 983.5]=>7/16 (43.7%)
  - IND5000: [0, 0, 0, 0, 821.5, 1151.5]=>4/12 (33%)
  - MAF 001 - 005: [0, 0, 0, 0]=>0/8 (0%)
  - MAF 005 - 02: [0, 0, 0, 0, 0, 0, 199.5, 949]=>3/16 (18.7%)
  - MAF 02 - 05: [391.5, 821.5, 983.5, 1151.5]=>8/8 (100%)
- Matrix Epistasis:
  - SNP1000: [1.5, 2]=>4/4 (100%)
  - SNP2500: [1.5, 1.5, 50, 76, 146.5, 492.5, 1027.5, 1038.5]=>16/16 (100%)
  - SNP5000: [2, 4.5, 10, 31, 32, 351]=>12/12 (100%)
  - IND1000: [1.5, 1027.5]=>4/4 (100%)
  - IND2500: [1.5, 1.5, 2, 50, 76, 146.5, 492.5, 1038.5]=>16/16 (100%)
  - IND5000: [2, 4.5, 10, 31, 32, 351]=>12/12 (100%)
  - MAF 001 - 005: [4.5, 31, 50, 492.5]=>8/8 (100%)
  - MAF 005 - 02: [1.5, 1.5, 1.5, 2, 2, 10, 1027.5, 1038.5]=>16/16 (100%)
  - MAF 02 - 05: [32, 76, 146.5, 351]=>8/8 (100%)
- MDR (discretized threshold #1):
  - SNP1000: [253, 253]=>2/4 (50%)
  - SNP2500: [0, 0, 3.5, 5, 252, 253, 257, 263.5]=>8/16 (50%)
  - SNP5000: [0, 0, 252, 252, 252.5, 256.5]=>4/12 (33.3%)
  - IND1000: [252, 263.5]=>2/4 (50%)
  - IND2500: [0, 0, 3.5, 5, 253, 253, 253, 257]=>8/16 (50%)
  - IND5000: [0, 0, 252, 252, 252.5, 256.5]=>4/12 (33%)
  - MAF 001 - 005: [0, 0, 0, 0]=>0/8 (0%)
  - MAF 005 - 02: [252, 252, 253, 253, 253, 256.5, 257, 263.5]=>8/16 (50%)
  - MAF 02 - 05: [3.5, 5, 252, 252.5]=>6/8 (75%)
- MDR (discretized threshold #2):
  - SNP1000: [0, 0]=>0/4 (0%)
  - SNP2500: [0, 0, 0, 0, 0, 0, 0, 0]=>0/16 (0%)
  - SNP5000: [0, 0, 0, 0, 0, 0]=>0/12 (0%)
  - IND1000: [0, 0]=>0/4 (0%)
  - IND2500: [0, 0, 0, 0, 0, 0, 0, 0]=>0/16 (0%)
  - IND5000: [0, 0, 0, 0, 0, 0]=>0/12 (0%)
  - MAF 001 - 005: [0, 0, 0, 0]=>0/8 (0%)
  - MAF 005 - 02: [0, 0, 0, 0, 0, 0, 0, 0]=>0/16 (0%)
  - MAF 02 - 05: [0, 0, 0, 0]=>0/8 (0%)
- MIDESP:
  - SNP1000: [0, 0]=>0/4 (0%)
  - SNP2500: [0, 0, 0, 0, 0, 0, 4154.5, 4996.5]=>4/16 (25%)
  - SNP5000: [0, 0, 0, 0, 0, 5419]=>1/12 (8.3%)
  - IND1000: [0, 0]=>0/4 (0%)
  - IND2500: [0, 0, 0, 0, 0, 0, 4154.5, 4996.5]=>4/16 (25%)
  - IND5000: [0, 0, 0, 0, 0, 5419]=>1/12 (8.3%)
  - MAF 001 - 005: [0, 0, 0, 5419]=>1/8 (12.5%)
  - MAF 005 - 02: [0, 0, 0, 0, 0, 0, 4154.5, 4996.5]=>4/16 (25%)
  - MAF 02 - 05: [0, 0, 0, 0]=>0/8 (0%)
- Plink BOOST (discretized threshold #1):
  - SNP1000: [1.5, 1.5]=>4/4 (100%)
  - SNP2500: [0, 0, 0, 1.5, 1.5, 1.5, 2, 2]=>10/16 (62.5%)
  - SNP5000: [0, 0, 1.5, 1.5, 1.5, 2]=>8/12 (66.7%)
  - IND1000: [0, 1.5]=>2/4 (50%)
  - IND2500: [0, 0, 1.5, 1.5, 1.5, 1.5, 2, 2]=>12/16 (75%)
  - IND5000: [0, 0, 1.5, 1.5, 1.5, 2]=>8/12 (66.7%)
  - MAF 001 - 005: [0, 0, 0, 0]=>0/8 (0%)
  - MAF 005 - 02: [0, 1.5, 1.5, 1.5, 1.5, 1.5, 2, 2]=>14/16 (87.5%)
  - MAF 02 - 05: [1.5, 1.5, 1.5, 2]=>8/8 (100%)
- Plink BOOST (discretized threshold #2):
  - SNP1000: [1.5, 1.5]=>4/4 (100%)
  - SNP2500: [0, 0, 1.5, 1.5, 1.5, 1.5, 2, 2.5]=>10/16 (62.5%)
  - SNP5000: [0, 0, 1.5, 1.5, 1.5, 3.5]=>8/12 (66.7%)
  - IND1000: [1.5, 1.5]=>2/4 (50%)
  - IND2500: [0, 0, 1.5, 1.5, 1.5, 1.5, 2, 2.5]=>12/16 (75%)
  - IND5000: [0, 0, 1.5, 1.5, 1.5, 3.5]=>8/12 (66.7%)
  - MAF 001 - 005: [0, 0, 0, 0]=>0/8 (0%)
  - MAF 005 - 02: [1.5, 1.5, 1.5, 1.5, 1.5, 1.5, 2.5, 3.5]=>14/16 (87.5%)
  - MAF 02 - 05: [1.5, 1.5, 1.5, 2]=>8/8 (100%)
- Plink Epistasis:
  - SNP1000: [1.5, 2]=>4/4 (100%)
  - SNP2500: [1.5, 1.5, 50, 76, 146.5, 492.5, 1026, 1038.5]=>16/16 (100%)
  - SNP5000: [2, 4.5, 10, 31, 32, 351]=>12/12 (100%)
  - IND1000: [1.5, 1026]=>4/4 (100%)
  - IND2500: [1.5, 1.5, 2, 50, 76, 146.5, 492.5, 1038.5]=>16/16 (100%)
  - IND5000: [2, 4.5, 10, 31, 32, 351]=>12/12 (100%)
  - MAF 001 - 005: [4.5, 31, 50, 492.5]=>8/8 (100%)
  - MAF 005 - 02: [1.5, 1.5, 1.5, 2, 2, 10, 1026, 1038.5]=>16/16 (100%)
  - MAF 02 - 05: [32, 76, 146.5, 351]=>8/8 (100%)
- QMDR:
  - SNP1000: [0, 251]=>1/4 (25%)
  - SNP2500: [0, 0, 0, 0, 0, 10, 251, 251.5]=>4/16 (25%)
  - SNP5000: [0, 0, 0, 0, 0, 0]=>0/12 (0%)
  - IND1000: [0, 10]=>2/4 (50%)
  - IND2500: [0, 0, 0, 0, 0, 251, 251, 251.5]=>3/16 (18.7%)
  - IND5000: [0, 0, 0, 0, 0, 0]=>0/12 (0%)
  - MAF 001 - 005: [0, 0, 0, 251]=>1/8 (12.5%)
  - MAF 005 - 02: [0, 0, 0, 0, 0, 10, 251, 251.5]=>4/16 (25%)
  - MAF 02 - 05: [0, 0, 0, 0]=>0/8 (0%)
- REMMA:
  - SNP1000: [2, 2]=>4/4 (100%)
  - SNP2500: [1.5, 1.5, 2, 90.5, 174, 969, 1570, 3544.5]=>16/16 (100%)
  - SNP5000: [2, 2.5, 8.5, 8.5, 77, 416]=>12/12 (100%)
  - IND1000: [2, 3544.5]=>4/4 (100%)
  - IND2500: [1.5, 1.5, 2, 2, 90.5, 174, 969, 1570]=>16/16 (100%)
  - IND5000: [2, 2.5, 8.5, 8.5, 77, 416]=>12/12 (100%)
  - MAF 001 - 005: [2.5, 77, 90.5, 969]=>8/8 (100%)
  - MAF 005 - 02: [1.5, 2, 2, 2, 2, 8.5, 1570, 3544.5]=>16/16 (100%)
  - MAF 02 - 05: [1.5, 8.5, 174, 416]=>8/8 (100%)

# SECTION 10 TRUE POSITIVE RATE BY INTERACTION ALPHA AND PURITY

For this analysis only regular dominant, multiplicative, recessive, and XOR datasets were used, that is the large dominant datasets were excluded.

- EpiSNP:
  - IA 1.25: 0/22 (0%)
  - IA 1.5: 1/38 (2.6%)
  - IA 2: 1/22 (4.5%)
  - IA 3: 0/16 (0%)
  - IA 8: 4/12 (33.3%)
  - IA 16: 4/12 (33.3%)
  - Pure: 7/56 (12.5%)
  - Impure: 3/56 (5.3%)
- Matrix Epistasis:
  - IA 1.25: 0/22 (0%)
  - IA 1.5: 7/38 (18.4%)
  - IA 2: 10/22 (45.4%)
  - IA 3: 3/6 (50%)
  - IA 8: 9/12 (75%)
  - IA 16: 9/12 (75%)
  - Pure: 23/56 (41.1%)
  - Impure: 15/56 (26.8%)
- MDR (Threshold #1):
  - IA 1.25: 8/22 (36.4%)
  - IA 1.5: 23/38 (60.5%)
  - IA 2: 15/22 (68.2%)
  - IA 3: 5/6 (83.3%)
  - IA 8: 5/12 (41.7%)
  - IA 16: 7/12 (58.3%)
  - Pure: 38/56 (67.8%)
  - Impure: 25/56 (44.6%)
- MDR (Threshold #2):
  - IA 1.25: 11/22 (50%)
  - IA 1.5: 25/38 (65.8%)
  - IA 2: 16/22 (72.7%)
  - IA 3: 5/6 (83.3%)
  - IA 8: 3/12 (25%)
  - IA 16: 4/12 (33.3%)
  - Pure: 40/56 (71.4%)
  - Impure: 24/56 (42.8%)
- MIDESP:
  - IA 1.25: 5/22 (22.7%)
  - IA 1.5: 18/38 (47.4%)
  - IA 2: 11/22 (50%)
  - IA 3: 5/6 (83.3%)
  - IA 8: 3/12 (25%)
  - IA 16: 1/12 (8.3%)
  - Pure: 27/56 (48.2%)
  - Impure: 16/56 (28.6%)
- BOOST (Threshold #1):
  - IA 1.25: 1/22 (4.5%)
  - IA 1.5: 2/38 (5.3%)
  - IA 2: 4/22 (1.8%)
  - IA 3: 0/6 (0%)
  - IA 8: 1/12 (8.3%)
  - IA 16: 1/12 (8.3%)
  - Pure: 6/56 (10.7%)
  - Impure: 3/56 (5.3%)
- BOOST (Threshold #2):
  - IA 1.25: 1/22 (4.5%)
  - IA 1.5: 0/38 (0%)
  - IA 2: 6/22 (27.2%)
  - IA 3: 0/6 (0%)
  - IA 8: 2/12 (16.7%)
  - IA 16: 1/12 (8.3%)
  - Pure: 5/56 (8.9%)
  - Impure: 5/56 (8.9%)
- Plink:
  - IA 1.25: 0/22 (0%)
  - IA 1.5: 7/38 (18.4%)
  - IA 2: 10/22 (45.4%)
  - IA 3: 3/6 (50%)
  - IA 8: 9/12 (75%)
  - IA 16: 9/12 (75%)
  - Pure: 23/56 (41.1%)
  - Impure: 15/56 (26.8%)
- QMDR:
  - IA 1.25: 6/22 (27.2%)
  - IA 1.5: 17/28 (60.1%)
  - IA 2: 10/22 (45.4%)
  - IA 3: 2/6 (33.3%)
  - IA 8: 4/12 (33.3%)
  - IA 16: 3/12 (25%)
  - Pure: 31/56 (55.3%)
  - Impure: 11/56 (19.6%)
- REMMA:
  - IA 1.25: 1/22 (4.5%)
  - IA 1.5: 12/38 (31.6%)
  - IA 2: 13/22 (59.1%)
  - IA 3: 3/6 (50%)
  - IA 8: 9/12 (75%)
  - IA 16: 9/12 (75%)
  - Pure: 28/56 (50%)
  - Impure: 19/56 (33.9%)

# SECTION 11 ASSEMBLY OF TOOLS

Seeing as each tool has its own strengths and weaknesses it is natural to ask whether the tools can be somehow combined in order to attain better overall performance than from any single tool.

As shown above each tool has its own strength and weaknesses.

MDR seems to be most versatile, while EpiSNP is least effective, with remaining tools performing somewhere in between.

One way to take advantage of multiple tools is by using them together. An effective scheme could be such that a detection made by at least two tools (using different underlying models) is given more weight. Especially, if this detection is near the top of the ranked list for multiple tools.

If looking for interactions of pre-determined statistical type (ex: dominant), an appropriate tool could also be selected in a targeted manner.
